# Systematic Identification of Essential Genes Required for Yeast Cell Wall Integrity: Involvement of the RSC Remodelling Complex

**DOI:** 10.3390/jof8070718

**Published:** 2022-07-08

**Authors:** Ana Belén Sanz, Sonia Díez-Muñiz, Jennifer Moya, Yuliya Petryk, César Nombela, José M. Rodríguez-Peña, Javier Arroyo

**Affiliations:** Departamento de Microbiología y Parasitología, Facultad de Farmacia, Universidad Complutense de Madrid, Instituto Ramón y Cajal de Investigaciones Sanitarias (IRYCIS), 28040 Madrid, Spain; absanzsa@ucm.es (A.B.S.); soniadiez@farm.ucm.es (S.D.-M.); jmoyavaquero@gmail.com (J.M.); yupetryk@ucm.es (Y.P.); cnombela@ucm.es (C.N.); jarroyo@ucm.es (J.A.)

**Keywords:** cell wall, stress response, screening, transcription, yeast, essential genes

## Abstract

Conditions altering the yeast cell wall lead to the activation of an adaptive transcriptional response mainly governed by the cell wall integrity (CWI) mitogen-activated protein kinase (MAPK) pathway. Two high-throughput screenings were developed using the yTHC collection of yeast conditional mutant strains to systematically identify essential genes related to cell wall integrity, and those required for the transcriptional program elicited by cell wall stress. Depleted expression of 52 essential genes resulted in hypersensitivity to the dye Calcofluor white, with chromatin organization, Golgi vesicle transport, rRNA processing, and protein glycosylation processes, as the most highly representative functional groups. Via a flow cytometry-based quantitative assay using a CWI reporter plasmid, 97 strains exhibiting reduced gene-reporter expression levels upon stress were uncovered, highlighting genes associated with RNA metabolism, transcription/translation, protein degradation, and chromatin organization. This screening also led to the discovery of 41 strains displaying a basal increase in CWI-associated gene expression, including mainly putative cell wall-related genes. Interestingly, several members of the RSC chromatin remodelling complex were uncovered in both screenings. Notably, Rsc9 was necessary to regulate the gene expression of CWI-related genes both under stress and non-stress conditions, suggesting distinct requirements of the RSC complex for remodelling particular genes.

## 1. Introduction

The fungal cell wall is an essential structure responsible for cell morphology; it contributes to preserving osmostability, protects cells against environmental stresses, and delineates cellular immunogenicity. In the eukaryotic model *Saccharomyces cerevisiae*, the cell wall consists of three major components: an inner layer of glucans (β-1,3 and β-1,6-glucan); a small amount of chitin (polymer of N-acetylglucosamine) and an outer layer of mannoproteins (such as agglutinins and flocculins involved in cell adhesion; and others related to enzymatic or structural functions). All these components must be correctly assembled to build a functional structure (for review, see [1,2]). Despite its apparent rigidity, the cell wall is very dynamic, so its composition and organization vary during growth and development. Additionally, when yeast cells face conditions where the cell wall integrity is compromised an adaptive response is triggered to maintain cell viability. This response includes changes in the synthesis and crosslinking of different polymers, and increased expression levels of genes functionally related to cell wall maintenance [3,4]. Cell wall stress adaptive responses in yeast are mainly regulated by the cell wall integrity (CWI) MAPK pathway, which includes a conserved MAPK module [5,6]. This module is activated through a cascade of phosphorylation events, which ultimately lead to the phosphorylation of the MAPK of the pathway, Slt2/Mpk1. In turn, phosphorylated Slt2 activates the transcription factor Rlm1, the main factor responsible for the transcriptional reprogramming elicited under these conditions [7,8,9]. CWI signalling is triggered by a variety of cell wall stressors (such as cell wall-interfering compounds) [4] or mutations in genes affecting proteins involved in cell wall homeostasis. Yeast mutants deleted for functionally relevant cell wall-related genes display constitutive Slt2 hyperactivation [10,11]. It was recently claimed that nucleosome remodelling at the promoters of Rlm1-dependent genes through cooperation between the ATP-dependent chromatin-remodelling SWI/SNF and acetyltransferase SAGA complexes is required for the binding of phosphorylated Rlm1, to target promoters and assembly of the transcription initiation machinery under cell wall stress [9,12]. In fact, mutants in different SWI and SAGA subunits display cell wall-related phenotypes. Additionally, various works also reported the association of cell wall integrity with mutants in some subunits of the RSC (Remodel the Structure of Chromatin) complex [13,14,15,16], which belongs to the SWI/SNF family of ATP-dependent chromatin remodellers [17]. However, information is lacking regarding their possible connection with CWI-dependent genes.

Largely due to its potential as a selective target for antifungal drugs, in addition to other biotechnological purposes, a great effort has been made to characterize genes involved in biogenesis, regulation, and maintenance of the yeast cell wall. This objective was fuelled by the availability of the complete collection of haploid yeast mutant strains individually deleted in each of the approximately 4800 non-essential genes identified in this organism [18]. To date, several genome-wide phenotypic or chemical-genomic profiling screens have been conducted using this yeast knock-out collection to identify cell wall-related mutants. Hypersensitivity to K1 and K2 killer toxins that form lethal pores in the plasma membrane [19,20], Congo red (chitin-binding dye), caspofungin (β-1,3-glucan synthase inhibitor), zymolyase (enzymatic cocktail including β-1,3-glucanase and chitinase activities) [21,22], freeze-thaw or heat stress [23,24] are some examples of the screenings developed to date. These experiments have permitted the identification of a broad functional catalogue of genes potentially related to maintaining cellular integrity under cell wall stress conditions. Moreover, from their comparative analysis, it has been possible to infer damage-specific signalling mechanisms and uncover genes required to withstand both specific and general cell wall injuries.

Following a complementary approach, we performed large-scale screenings using the whole collection of haploid deletion strains in all non-essential genes of *S. cerevisiae* transformed with a reporter system based on transcriptional fusion of the *MLP1*/*KDX1* (*YKL161C*) promoter to the coding sequence of the *nat1* gene encoding nourseothricin N-acetyltransferase from *Streptomyces noursei* [25]. Firstly, we used this collection of mutant strains to identify genes whose absence produces constitutive activation of the CWI pathway under non-stress growth conditions [11]. Globally, this strategy uncovered mutants related to signalling and potential effectors required to maintain cell viability upon cell wall stress. Additionally, we used the same collection in a second screening to identify elements involved in the adaptive transcriptional response elicited upon cell wall stress and required for proper gene expression under stress conditions affecting cellular integrity [9]. This approach allowed the identification of several protein complexes related to the regulation of gene expression upon zymolyase-mediated cell wall damage conditions, including the SWI/SNF ATP-dependent chromatin-remodelling complex.

All these functional studies did not include the approximately one thousand yeast genes essential for viability under standard laboratory growth conditions, which account for nearly 19% of the genes identified in the *S. cerevisiae* genome [18,26]. Considering that around 20% of yeast genes may show a direct or indirect functional relationship with cell wall homeostasis [27], and the fact that this structure is essential for cell survival, this functional group would be expected to be significantly represented within the group of essential genes.

In practice, functional genomics applied to essential genes is scarce due to the difficulty of handling these yeast strains. In this context, several strategies have been developed to facilitate these studies, particularly temperature-sensitive mutations or conditionally degradable fusion proteins containing a degron sequence [28]. These approaches show, as the main drawbacks, that repression conditions can affect cell physiology (heat shock) and sequence alteration of the ORFs under study. As an alternative, the yeast Tet-promoters Hughes collection (yTHC) [29] is available. This collection, which has been successfully used in several large-scale screens [28] including those related to MAPK signalling [30], encompasses approximately 75% of essential yeast genes. It is based on replacing the native promoter region of essential genes with the tetracycline (tet)-regulatable promoter to create mutant conditional alleles. In this way, repression of expression is controlled by adding doxycycline to the growth medium, without affecting yeast physiology.

Using the yTHC collection, we developed two different types of screenings to identify cell wall-related genes. One aimed to identify essential genes required to cope with cell wall damage conditions imposed by treatment with the chitin-binding dye Calcofluor white (CW). The second screening was designed to identify elements required for the adaptive transcriptional response induced by cell wall stress. Using both strategies, we uncovered yeast genes related to cell wall maintenance, including those associated with the biogenesis of this structure and the regulatory elements required for this process. We have delved deeper into the study of the participation of the RSC chromatin remodelling complex in regulating the expression of CWI pathway-dependent genes. All this information increases our basic knowledge of the gene catalogue required to withstand cell wall damage. In addition, it will facilitate the establishment of the mode of action of antifungal drugs that target products encoded by essential genes.

## 2. Materials and Methods

### 2.1. Yeast Strains and Media

All the experiments in this work were performed using the *Saccharomyces cerevisiae* Tet-promoters collection (yTHC) from the Hughes Laboratory, University of Toronto (Canada), provided by Open Biosystems (Dharmacon Inc., GE Healthcare, UK). It includes 821 essential yeast genes whose expression is regulated by doxycycline, since in each strain the endogenous promoter has been replaced with the TetO_7_ promoter. This collection was created using the strain R1158 (BY4741 background) *URA3::CMV-t*TA *MATa his3-1 leu2-0 met15-0*.

Yeast strains were grown on YPD medium (2% glucose, 2% peptone, 1% yeast extract). For selection of yeast transformants bearing the pBS1 plasmid, SD-His medium was used (0.17% yeast nitrogen base, 0.5% ammonium sulphate, 2% glucose, supplemented with the required amino acids). When required, doxycycline (Calbiochem, Darmstadt, Germany) was added to the culture medium at a final concentration of 10 μg/mL.

### 2.2. Calcofluor White Sensitivity Screening

The complete yTHC collection, including the wild-type strain, was grown in 96-well microtiter plates in 200 μL of YDP medium for 24–48 h at 30 °C. Afterwards, 5 μL of these cultures (optical density ~0.15 at 595 nm) were inoculated in a new set of four 96-well microtiter plates containing 145 μL of YPD, YPD plus 10 μg/mL of doxycycline (DOX), YPD plus 40 μg/mL of Calcofluor white (CW, Fluorescent brightener 28, Sigma-Aldrich, Merck, Darmstadt, Germany) and YPD+DOX+CW. After 48 h of static incubation at 30 °C, the level of growth was determined by measuring the absorbance at 595 nm in each well using a microplate reader (Model 680, Bio-Rad, Hercules, CA, USA). The level of sensitivity of each mutant was calculated as follows. First, we obtained the absorbance ratios after growing in YPD+DOX+CW vs. YPD+CW and YPD+DOX vs. YPD (as an indicator of non-CW mediated effects). Finally, both ratios were divided (presence vs. absence of CW) to obtain a numerical value corresponding to the level of sensitivity of each mutant allele to CW when gene expression is blocked in the presence of doxycycline. After an initial global screening, a mutant strain was considered hypersensitive to the drug when this final ratio was ≤0.2. To focus on mutants hypersensitive to CW when gene expression is turned off by doxycycline, those mutants largely affected in growth in the presence of the drug in the absence of doxycycline were not further considered. Finally, those mutants selected from the first screening (CW hypersensitive) were retested in two additional independent sensitivity assays as described above. Eventually, those mutants showing mean ratios ≤ 0.2 were selected as positive hits of the screening.

### 2.3. Screening for Altered MLP1-GFP Expression Using Flow Cytometry

Initially, the full collection of Tet-promoter mutant strains was individually transformed following the lithium acetate protocol with the pBS1 plasmid using SC-His plates as selection medium. This plasmid is a variant of the *MLP1pro-MLP1-GFP* plasmid [25] in which the *Sma*I-digested *HIS3* ORF (1.7 Kb) from plasmid p34H-*HIS3* [31] was introduced into the *Stu*I site within the *URA3* gene, thus changing the selection marker. To identify mutants showing alterations in *MLP1* expression upon cell wall stress, one transformant from each mutant strain was grown in YPD medium supplemented with 10 μg/mL of doxycycline at 24 °C overnight and then divided into two parts. One part continued growing under the same conditions (non-treated culture), whereas the other was supplemented with CW (10 μg/mL). Cells were harvested after 3 h of incubation, washed twice with PBS, and finally resuspended in PBS supplemented with 0.4 μg/mL propidium iodide (PI) (Sigma Aldrich, St. Louis, MO, USA) and analysed using a FACSCan flow cytometer (Becton Dickinson, Bergen County, NJ, USA) equipped with an argon-ion laser emitting at 488 nm by acquiring green fluorescence emission through a 530/30 bandpass (BP) filter. For each sample, the mean fluorescence intensities (MFIs) and the percentage of GFP-positive cells within the PI-negative population (dead cells are stained with PI) were obtained using the BD CellQuest 3.3 software (Becton Dickinson) on Mac^®^ OS 9.2.2. Following this, the MFI ± CW ratio was calculated for each mutant strain (R1) and the wild-type strain (R2). Finally, those mutants displaying a quotient R1/R2 ≤ 0.25 and/or a percentage of GFP-positive cells in the presence of CW lower than 15% (mean value for the wild-type strain is around 75%) were initially selected as positive hits from the screening. To confirm these data, these mutants were retested, as described above, using two additional independent transformants. Eventually, those mutants showing a mean MFI R1/R2 from the three replicates ≤0.25 and/or lower than 15% of GFP-positive cells (as mean value) were considered positive hits.

### 2.4. Western Blotting Assays

The procedures used for immunoblotting analyses, including cell collection and lysis, collection of proteins, fractionation by SDS–PAGE, and transfer to nitrocellulose membranes were performed as previously described [32] using the Odyssey Infrared Imaging System (LI-COR). The detection of phosphorylated Slt2 was accomplished using the anti-phospho-p44/p42 MAPK monoclonal antibody (thr202/tyr204; Cat. No. 4370; Cell Signaling Technology, Beverly, MA, USA). To monitor protein loading, Glucose-6-phosphate dehydrogenase levels were determined using an anti-G6PDH polyclonal antibody (Cat. No. A9521; Sigma-Aldrich Corp, St. Louis, MO, USA). The secondary antibodies used were IRDye 800CW goat anti-rabbit (Cat. No. 926-32211) and IRDye 680LT goat anti-mouse (Cat. No. 926-68020), both from LI-COR Biosciences (Lincoln, NE, USA). All antibodies were used at the dilutions recommended by the manufacturers. Protein band quantification was carried out by densitometric analysis using the software Image Studio Lite (LI-COR Biosciences). Phospho-Slt2 protein levels were normalized against the loading control.

### 2.5. Quantitative RT-PCR Assays

RNA isolation and RT-qPCR assays were performed as previously described [8]. For quantification, the abundance of each transcript was determined using the amount of the standard transcript *ACT1* for input cDNA normalization, and final data on relative gene expression between the conditions tested were calculated following the 2^−ΔΔCt^ method [33]. Oligonucleotide sequences are available upon request.

## 3. Results and Discussion

### 3.1. High-Throughput Screening for Essential Genes Required to Cope with Cell Wall Damage

**Figure 1:**
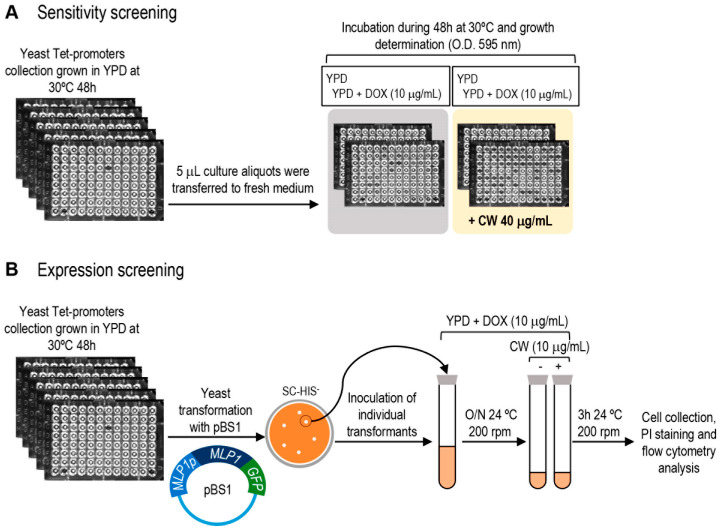
Overview of the screening strategies designed to identify cell wall-related mutants in essential genes. (**A**) Hypersensitive phenotype to Calcofluor white was studied in a 96-well microtiter plate format. (**B**) Detection of altered expression levels of the CWI-reporter gene *KDX1*/*MLP1*. The complete yeast Tet-promoter collection was transformed with the plasmid pBS1 (*MLP1p-MLP1-GFP*) and individual transformants were tested for Mlp1-GFP expression in the presence or absence of Calcofluor white (CW) by flow cytometry. DOX: exposure to doxycycline at the indicated concentration. PI: Propidium Iodide.

We designed a screening using the yTHC collection of yeast conditional mutant strains containing 821 of the 1105 total reported essential genes to obtain the complete repertoire of essential genes related to cell wall maintenance. The rationale of this screening was that those strains with a defective cell wall would display a phenotype of hypersensitivity to agents that disturb this essential fungal structure [27]. To identify strains hypersensitive to the cell wall-interfering compound CW, under restrictive conditions (repression of gene expression due to the presence of doxycycline in the culture medium), we first set up the optimal growth conditions for the yeast collection in a 96 multi-well plate format using sublethal concentrations of CW. Eventually, the assay was performed at static incubation at 30 °C, in the presence or not of 10 μg/mL of doxycycline and 40 μg/mL CW. The effect on cell growth was quantified by measuring the optical density at 595 nm after 48 h (see Figure 1). As explained in detail in Materials and Methods, the mutant strains were considered hypersensitive to CW when their growth was reduced by this agent in the presence of doxycycline in the culture medium in comparison with the same conditions but in the absence of CW. Following this approach, for each conditional allele we obtained a growth ratio (equivalent to sensitivity ratio) from 0 (maximum sensitivity due to down-regulation of gene expression) to ~1 (absence of effect on growth, as observed for the wild-type strain), indicative of the relative hypersensitivity level to CW (Figure 1 and Table S1). The mutant strains that exhibited higher sensitivity levels (arbitrary cut-off growth ratio ≤ 0.2) were selected as preliminary positive hits from this first round of screening. Next, this group of mutants underwent two additional screening rounds under the same experimental conditions to finally select those with a reproducible CW hypersensitivity phenotype (see Material and Methods for details). Finally, 52 mutant strains were considered positive hits of the screening (Table 1 and Table S2). The CW hypersensitivity of some selected mutants was validated by serial dilution assays in the presence or absence of doxycycline (Figure S1).

To determine the functional categories of the genes selected in the screening, we manually grouped the gene set using the functional annotation deposited in the *Saccharomyces Genome Database* (SGD) tool “GO Slim Mapper: process”. For the 52 CW hypersensitive mutants, the more represented groups corresponded to chromatin organization (8), Golgi vesicle transport (8), rRNA processing (8), and protein glycosylation/glycosylphosphatidylinositol (GPI) anchor biosynthesis (7) (Table 1). In other cell wall-related screenings carried out on the complete collection of viable haploid yeast deletion mutants, similar functional groups were uncovered [22,34]. Moreover, these screenings including treatments with agents interfering with cell-wall construction via different mechanisms of action, such as Congo red, zymolyase, or caspofungin, uncovered a high percentage of specific mutants for each drug [22]. We can speculate that the situation may be similar in the case of mutants in essential genes. In any case, additional work will be necessary, using alternative cell-wall interfering compounds to confirm this hypothesis.

It is reasonable to speculate that some of the mutants identified in our screening could be ascribed to the so-called “Type I” of essential genes, which are those postulated to execute “core” functions indispensable to the organism [35], like those included in the group of rRNA processing. The hypersensitivity to cell wall interfering agents, in this case, could be somehow associated with pleiotropic effects on cell wall architecture. However, other genes seem more directly related to cell wall biogenesis or maintenance, such as those involved in protein glycosylation or GPI biosynthesis, which are required for the correct function of cell wall proteins or those related to vesicle transport required for the homeostasis of this structure (see next sections). In addition, recent studies have shown that the MAPK CWI signalling pathway regulates the expression of genes necessary for survival in the presence of agents that damage the cell wall in collaboration with the chromatin remodelling complexes SWI/SNF [9] and SAGA [12]. In fact, we found components of SWI/SNF (*ARP7*) and SAGA (*TAF5* and *TAF10*), and, remarkably, we also identified many members of the RSC chromatin remodelling complex (*ARP7*, *RSC6*, *RSC8*, *RSC58*, and *STH1*). This result implies that the RSC complex plays an important role under cell wall stress conditions. In this respect, previous studies have reported that some *rsc* mutants show cell wall-related phenotypes [13,14,15,16].

### 3.2. Identification of Essential Genes Required for the Transcriptional Adaptive Cell Wall Stress Response

In yeast, cell wall damage triggers a compensatory mechanism principally based on the transcriptional induction of a group of effector genes, finely regulated via the CWI pathway governed by the MAPK Slt2 [4]. Therefore, identifying essential genes involved in this process is of great interest to complete the catalogue of genes associated with this adaptive cellular response. To this end, complementary to the phenotypic study, we designed and developed a flow cytometry screening to discover yeast mutants for essential genes affected in the transcriptional induction caused by treatment with CW (Figure 1). The yTHC collection was individually transformed with a plasmid incorporating the fusion of the *MLP1*/*KDX1* gene coding sequence (encoding a pseudokinase paralog of Slt2) to that of the GFP protein, expressed under the control of the native *MLP1* promoter [25]. *MLP1* shows low basal gene expression levels but is highly expressed under cell wall stress, making this gene of particular usefulness for transcriptional studies. Moreover, it is controlled via the CWI pathway, and therefore its up-regulation is largely dependent on Slt2 and the transcription factor Rlm1 [8,25,36,37]. The wild-type strain of this collection (R1158 background) transformed with the plasmid containing the *MLP1-GFP* fusion presented, under non-stress conditions, 3.09 ± 1.92% of positive cells for GFP fluorescence with a mean fluorescence intensity (MFI) of 22.68 ± 3.5. Upon induction conditions (3 h exposure to CW), these values rose sharply to 73.69 ± 7.0% of GFP positive cells and MFI values around 154.50 ± 39.20. These data indicate that the *MLP1-GFP* construction offers optimum fluorescence levels to identify mutant strains affected in the expression of this CWI-reporter under basal and stress conditions. As an example, GFP fluorescence images of a wild-type and a mutant strain in which *MLP1* induction is blocked are shown (Figure S2).

**Figure 2:**
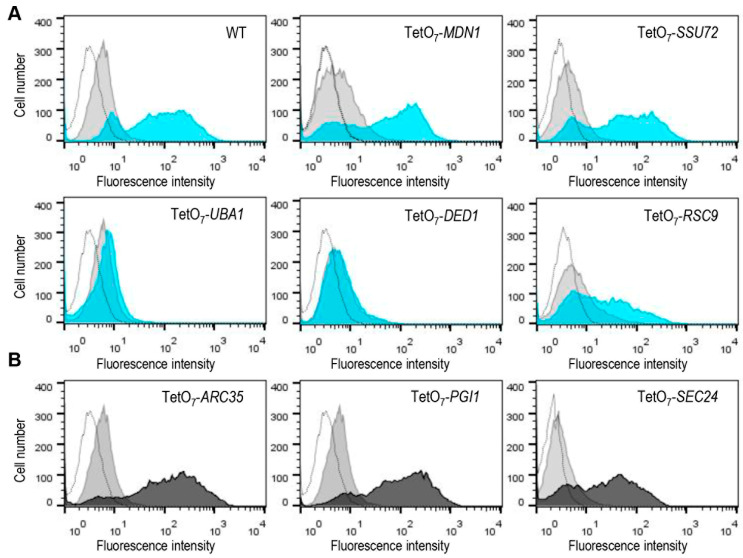
Identification of mutants from the yTHC collection that exhibit altered Mlp1-GFP levels by flow cytometry analysis. (**A**) Representative experiments of mutants in which Mlp1-GFP fluorescence levels were found to be affected (lower panels), or not affected (upper panels), compared with those of the wild-type strain. For each strain, histograms show the distribution of GFP fluorescence under non-stress conditions (grey-filled) and after treatment with Calcofluor white (10 μg/mL for 3 h) (blue-filled) in the presence of doxycycline (10 μg/mL). (**B**) Examples of mutants displaying increased GFP signal under non-stress conditions (absence of CW) in the presence of doxycycline are shown. For each conditional allele, histograms show basal GFP fluorescence corresponding to wild-type (grey-filled) and mutant (black-filled) strains handled in parallel. Control cells that do not express GFP are shown in each histogram overlay (unfilled peaks).

As described in Materials and Methods and illustrated in Figure 1, the complete collection of yeast mutants expressing Mlp1-GFP was grown in a rich medium including 10 µg/mL of doxycycline and then exposed, or not, to CW for 3 h. Next, GFP fluorescence levels, as the readout of Mlp1-GFP amounts for each mutant tested, were quantified by flow cytometry. The wild-type strain was always included as a control of *MLP1* induction in each experiment. Thus, the effect of the down-regulation of the expression of each conditional allele on Mlp1-GFP levels after CW treatment was determined, calculating first the ratio of MFIs between treatment and control conditions (absence of CW) for the wild-type and the mutant strains. Finally, the ratio obtained for each mutant strain was divided by that calculated for the wild-type strain. Flow cytometry data analyses uncovered mutants with changes in global MFIs and mutants in which the number of cells expressing detectable fluorescence signals was affected. Representative examples of the different flow cytometry patterns obtained are shown in Figure 2A. From these observations, in a first round of screening, we preliminarily selected those mutants showing at least a 75% decrease in Mlp1-GFP levels with respect to those of the wild-type strain (fluorescence ratio mutant/fluorescence ratio WT ≤ 0.25) and/or showing a percentage of GFP positive cells lower than 15% (Table S3). Next, two additional experiments were conducted using different yeast transformants of this group of mutants to finally define the positive hits of the screening (see Material and Methods for details and Table S4).

**Figure 3:**
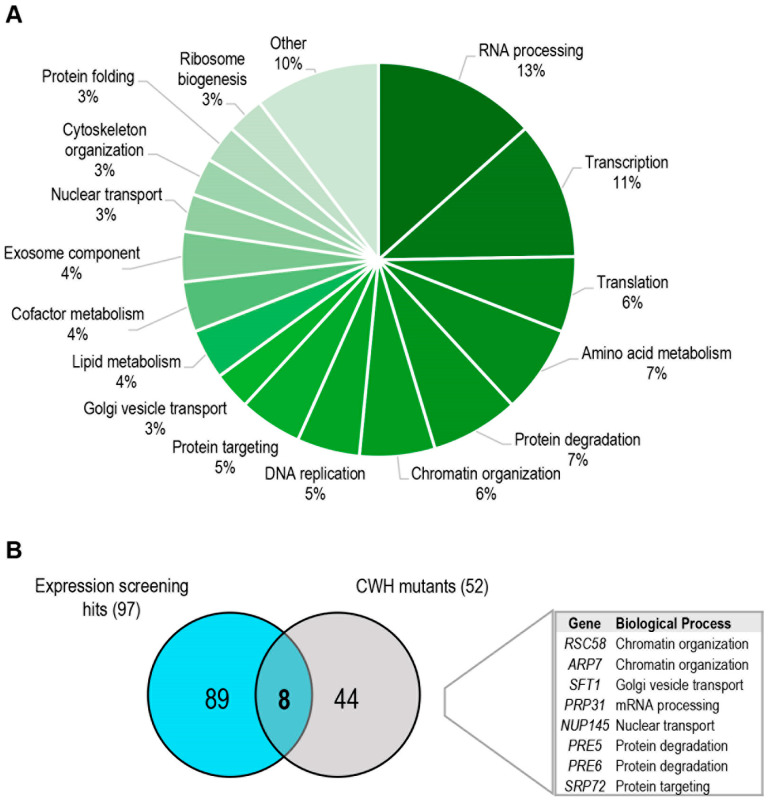
Functional classification of genes identified in the expression screening. (**A**) Relative distribution of functional categories based on Gene Ontology (biological process) from the 97 positive hits. (**B**) The common group of essential gene mutants uncovered in both the phenotypic and expression screenings are shown. CWH: Calcofluor white hypersensitive.

We sought functional categories within the group of 97 mutants selected (Figure 3A and Table S4). The most represented group (17%) corresponded to that associated with RNA metabolism, including RNA processing and degradation (exosome component). The identification of this group of genes, in conjunction with those associated with transcription, ribosome biogenesis, translation, and protein folding, could be explained as a consequence of alterations in the cellular machinery required for the correct expression and translation of the gene reporter Mlp1 under cell wall stress. However, it cannot be ruled out that CWI pathway signalling or regulatory defects could also be present in some mutants. Other groups of interest corresponded to those related to lipid metabolism, protein degradation, nucleocytoplasmic RNA transport, or chromatin remodelling. Within the first group, we found genes involved in ergosterol biosynthesis (*ERG1* and *ERG26*) or sphingolipid metabolism (*LCB2* and *TSC13*). Their identification is in accordance with the fact that sphingolipids are a structurally diverse class of lipids implicated in many cell signalling functions and interconnected with the ergosterol pathway [38]. The protein degradation group includes several subunits of the 26S proteasome (*PRE5*, *PRE6*, *RPN11*, and *RPT2*) and some ubiquitin-related enzymes (*UBA1*, *CDC34*, and *HRT1*). Interestingly, proteasome up-regulation has been proposed under different stress conditions, such as heat shock or oxidative stress [39,40]. Moreover, a control of proteasome abundance mediated by the MAPK Slt2 has also been reported [41]. Since proteasome-mediated degradation regulates the turnover of numerous cellular proteins involved in the majority of cellular processes [42], it is difficult, in these mutants, to discern between the existence of direct effects on CWI pathway regulation, mainly due to ubiquitination, or pleiotropic effects that indirectly alter the adaptive transcriptional response. Interestingly, proteasome inhibition leads to a slight increase in CW sensitivity (our unpublished results). Additional work will be required to clarify the molecular mechanisms involved in the CWI pathway-proteosome interaction. Still, the identification of only certain elements of this large protease complex allows us to hypothesize that they are particularly important under cell wall stress conditions.

Other mutants of interest are those related to nucleocytoplasmic RNA transport (*NUP145* or *MEX67*). Considering that, under heat shock stress, a selective nuclear export of heat-shock mRNAs and retention of regular transcripts mediated by Mex67 have recently been reported [43], similar or alternative mechanisms may take place under other stress conditions. Finally, it is important to highlight the presence of genes functionally related to chromatin remodelling, including essential subunits of the SWI/SNF (*ARP7*) and SAGA (*TAF9*) chromatin-modifying complexes, which were previously associated with the regulation of the expression of CWI-dependent genes upon cell wall stress [9,12]. Interestingly, within this group of chromatin-associated mutants, we also found essential elements of the RSC remodelling complex (*ARP7*, *RSC58*, and *RSC9*). In agreement, different ATP-dependent chromatin remodelling complexes work together to regulate the expression of stress-responsive genes [44,45].

Comparative analyses of the genes identified in both the phenotypic and transcriptional screenings under the imposed cut-off thresholds revealed a reduced number of mutants, including two subunits of the RSC complex and two proteasome members (Figure 3B). This low overlap agrees with previous studies with the complete collection of viable deletion mutants after zymolyase treatment. In these assays, 154 mutants exhibited hypersensitivity to zymolyase [22], and 159 mutants showed a reduction in the *MLP1*-reporter expression under zymolyase treatment [9], but only 35 mutants were found common in both studies. In agreement, most of the genes included in the cell wall damage compensatory mechanism work cooperatively to support cell wall integrity, but they are not essential individually to maintain cell viability under stress situations, except for those involved in signalling or transcriptional regulation, where the overall response is impaired. The elements identified of the RSC complex would belong to this category.

### 3.3. Impact of the RSC Complex on the Expression of CWI Pathway-Dependent Genes

We uncovered several elements of the RSC chromatin remodelling complexes showing CW hypersensitivity and/or alteration of Mlp1-GFP levels under cell wall stress conditions (Table S2 and Table S4). The RSC complex consists of 17 subunits (both essential and dispensable for cell viability) that can work in smaller subcomplexes, hindering its functional characterization [46]. This complex is mainly involved in transcription regulation, remodelling nucleosomes in promoter and transcribed coding sequences [47,48]. It also plays a role in chromosomal transactions such as DNA replication and repair and chromosome segregation [47,49]. In agreement with the functional complexity of RSC, previous large-scale transcriptional studies carried out under basal growth conditions of several yeast RSC subunit mutants (*rsc3*, *rsc30*, *rsc4* or *rsc14*) have demonstrated a small overlap in the genes regulated by the different subunits [13,50,51]. Additionally, in these studies, some RSC subunits modulated the expression of a reduced group of cell wall-related genes.

**Figure 4:**
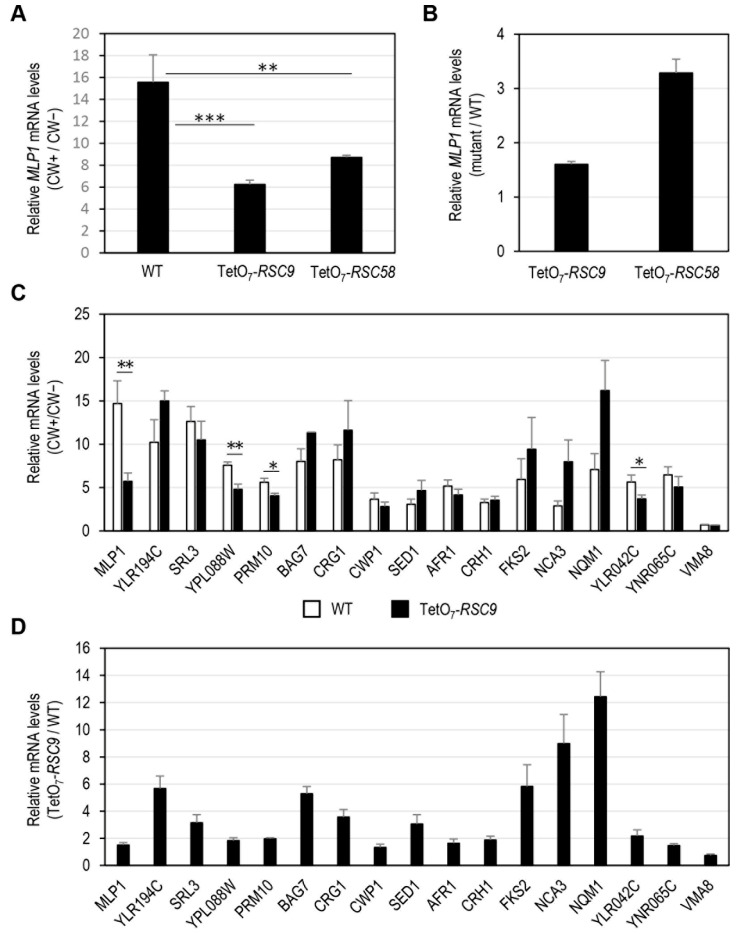
Gene expression analysis of CWI-responsive genes in the wild-type strain and mutants in essential subunits of the RSC complex. (**A**) *MLP1* mRNA levels of the wild-type (WT), TetO_7_-*RSC9*, and TetO_7_-*RSC58* strains treated with doxycycline (DOX, 10 μg/mL) and exposed or not to CW (10 μg/mL for 3 h) were analysed by RT-qPCR. Values represent the ratio between CW-treated and nontreated cells grown in the presence of doxycycline. (**B**) Basal mRNA levels of *MLP1* in TetO_7_-*RSC9* and TetO_7_-*RSC58* strains in the presence of DOX. Values represent the ratio between mutant and WT cells. (**C**) Analysis of mRNA levels of several selected CWI-responsive genes in the WT and TetO_7_-*RSC9* strains as indicated in A. A CWI-independent gene, *VMA8*, was included as a control. (**D**) Impact of depletion of Rsc9 on the basal expression levels of the indicated CWI-related genes, represented as indicated in panel B. Data correspond to the mean and standard deviation of at least three independent experiments. Statistical significance was determined using a two-tailed, unpaired, Student’s t-test, comparing it with data from the WT strain (* *p* ≤ 0.05, ** *p* ≤ 0.01, *** *p* ≤ 0.001).

We aimed to further investigate the relationship between possible alterations in the cell wall of some essential *rsc* mutants identified in our screening and modulation of the adaptive transcriptional response mediated by the CWI pathway when cell wall integrity is compromised. Firstly, mRNA levels of the CWI reporter gene *MLP1* were quantified via RT-qPCR in *rsc9* and *rsc58* mutants. These subunits belong to the substrate recruitment module (SRM) of the RSC complex [46]. In the case of Rsc9, this element has been involved both in the repression and activation of specific stress-regulated genes (i.e., hydrogen peroxide and rapamycin [52] or osmostress [53]). The transcriptional induction of *MLP1* in the presence of CW was significantly affected in these mutants under shut-off gene conditions (Figure 4A), validating the experimental approach developed in our screening and suggesting that the RSC complex regulates transcription upon cell wall stress. In the absence of stress, both subunits negatively regulate *MLP1* transcription, as inferred from the increase in the amount of *MLP1* transcripts in both mutants, particularly in the *rsc58* strain (Figure 4B). Next, we investigated the impact of *rsc* mutations on the expression of additional CWI-dependent genes. To this end, the mRNA levels of 15 other genes induced by cell wall stress [8,36,37,54,55] were analysed under the conditions indicated above, focusing on the *rsc9* mutant (Figure 4C). Interestingly, we observed two types of genes regarding the effect caused by the absence of Rsc9. One group, including *YPL088W*, *PRM10*, and *YLR042C*, showed a behaviour similar to *MLP1* in terms of the impact on their expression levels after treatment with CW compared with the up-regulation observed in the wild-type strain; and a second, more extensive group, in which no significant alteration in transcript levels was observed. Quantification of the transcript levels of these genes under non-stress conditions in the *rsc9* mutant relative to those of the wild-type strain showed a derepression of genes *YLR194C*, *SRL3*, *BAG7*, *CRG1*, *SED1*, *FKS2*, *NCA3* and *NQM1* in the absence of *RSC9* (Figure 4D). This basal regulation was not manifested in the case of the *VMA8* gene, which encodes a subunit of vacuolar ATPase non-transcriptionally regulated by cell wall stress. Therefore, our results indicate that the RSC complex regulates the gene expression of certain CWI-related genes both under stress and non-stress conditions. As described above, positive or negative transcriptional modulation of specific genes under different stress conditions, probably a consequence of their interaction with other regulators (repressors or activators), has been previously described for Rsc9 [52] and other RSC subunits [13,51]. Under basal (non-stress) conditions, RSC could also be involved in stabilizing the nucleosomes present at some CWI-dependent genes. Similarly, some UPR genes show high basal level expression when RSC is disrupted, indicating the presence of inherently altered chromatin in their absence [45]. Upon cell wall stress conditions, Slt2 phosphorylates and activates the transcription factor Rlm1 to recruit both elements to the promoters of CWI-responsive genes in complex with SWI/SNF [9]. SWI/SNF activity is necessary to evict nucleosomes positioned in this region and permit pre-initiation complex (PIC) assembly in cooperation with the SAGA complex, which mediates histone acetylation for nucleosome reorganization [6,12]. The results described here suggest that the RSC complex could cooperate with SWI/SNF and SAGA complexes for the chromatin remodelling necessary for the transcriptional activation of CWI-dependent genes upon stress. There seems to be distinct requirements of the RSC complex for remodelling particular genes in a stress-dependent manner. Thus, the influence of the RSC complex via its subunit Rsc9 on the expression levels of genes regulated by the CWI pathway shows a highly gene-specific regulatory pattern, probably a consequence of each particular promoter architecture, chromatin status, and interaction with the transcriptional machinery.

### 3.4. Identification of Essential Genes Associated with the Constitutive Activation of the Cell Wall Integrity Pathway

One of the objectives of this work was to uncover essential genes that could be directly or indirectly related to the presence of alterations in the fungal cell wall. On many occasions, they were revealed by the constitutive activation of the CWI route without the need to apply any type of external stimulus. Presumably, this type of mutant should be included among those that exhibited an increment in Mlp1-GFP levels in the absence of CW when the essential gene expression is blocked (presence of doxycycline). We arbitrarily ascribed to this group those mutants exhibiting a relevant level of MFI (higher than three-fold) with respect to the value obtained for the wild-type strain (Table 2 and Table S5). Representative flow cytometry profiles of these mutants are shown in Figure 2B. Eventually, 41 yeast strains met these criteria. After functional analyses, as described above, we found that approximately 68% belonged to four functional groups closely related to cell wall homeostasis: Protein glycosylation, Golgi vesicle transport, Glycosylphosphatidylinositol (GPI)-anchor assembly, and Cytoskeleton organization. GPI-anchored proteins (GPI-APs) are luminal secretory cargos that are attached by a post-translational glycolipid modification, the GPI anchor, to the plasma membrane and/or cell wall in yeast, consisting of a process conserved among eukaryotes [56]. A number of the GPI proteins in yeast serve enzymatic functions required for the biosynthesis and continuous shape adaptations of the cell wall, some seem to be structural elements of the cell wall, and others mediate cell adhesion [57]. The secretory pathway plays a key role in cell wall construction and remodelling, which explains the identification of this type of mutant in our screening. In fact, some of them, Sec24 and Sar1 belonging to the coat protein complex II (COPII), are involved in the specialized transport of GPI-APs to the ER [56]. The relationship between protein glycosylation and cell wall integrity is well established since most cell wall proteins are glycoproteins that have passed through the secretory pathway in transit to the cell wall. Thus, the isolation of this type of mutant is not unexpected given the importance of glycosylation for the proper conformation, localization, and function of cell wall proteins [58]. Regarding actin cytoskeleton-related genes, potential cell wall alterations could be a consequence of the requirement of this structure for correct spatiotemporal vesicular trafficking and cell wall assembly [59]. In accordance with the putative existence of alterations in the cell wall of mutants displaying incremented basal levels of Mlp1-GFP, about 17% were also selected in the screening for CW hypersensitivity (Table 2). In agreement with this idea, this value was significantly higher (50%) when a less strict cut-off point (0.4) was considered for CW hypersensitivity (Table 2).

**Figure 5:**
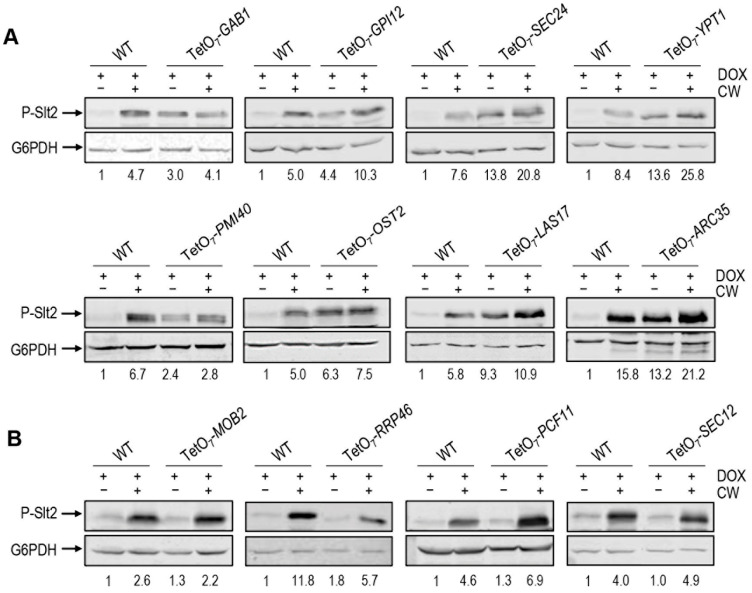
Detection of increased levels of Slt2 phosphorylation (P-Slt2) in the absence of cell wall stress. (**A**) Example of mutants displaying increased P-Slt2 basal levels. (**B**) Examples of mutants without P-Slt2 alteration. Cells exposed to doxycycline 10 μg/mL (DOX) treated or not with Calcofluor white 10 μg/mL for 3 h (CW) of the indicated yeast strains were taken and processed. Western blots detecting P-Slt2 with G6PDH as loading control are shown. Numbers correspond to the P-Slt2 fold-change obtained from densitometric quantification of the P-Slt2 bands normalized against G6PDH bands, using the values of the wild-type strain (WT) grown in the absence of CW as a reference (fold-change set to 1.0).

To further investigate whether the increase in Mlp1 expression in these mutants could be associated with the activation of the CWI pathway, we analysed the levels of phospho-Slt2 (typical readout of the level of activation of this pathway) in two mutants from each of the four functional groups described above. To this end, we obtained total protein extracts from these strains grown in the presence of doxycycline and treated or not with CW. Next, these extracts were analysed by Western blotting using an antibody that recognizes the dually phosphorylated (active) form of Slt2. As shown in Figure 5A, in the mutant strains tested, the levels of phospho-Slt2 under basal growth conditions were clearly higher than those observed in the wild-type strain. These results support the notion that the increase in Mlp1 levels detected in these mutants, when the conditional allele is off, is a consequence of the activation of the CWI pathway. Interestingly, as shown in Figure 5A, in the presence of CW, in some mutants the activation level (phospho-Slt2) does not increase beyond basal levels, while in others the ability to over-activate the pathway seems to be maintained. As expected, this effect was not observed in other mutants not affecting Mlp1-GFP levels under non-stress conditions (Figure 5B). Future gene-focused studies will be required to decipher whether the constitutive activation of the CWI pathway is due to signalling via the cell surface CWI pathway associated sensors or is mediated by downstream elements of the pathway, which eventually induces Slt2 phosphorylation.

Globally, it is noteworthy that the percentage of essential genes directly or indirectly related to cell wall integrity revealed in this work is similar to that predicted from pioneering cell wall phenotypic studies using viable knock-out mutant strains [27]; this supports the importance of maintaining the integrity of this fungal structure. The results described herein improve our knowledge regarding the putative connection between essential genes and cell wall integrity. This will greatly facilitate further in-depth studies directed towards investigating at which cellular level (signalling, transcriptional or post-transcriptional) a specific essential gene impacts on the adaptive response to withstand cell wall injuries. Moreover, the results of this work will be useful for the development of targeted studies on specific genes, particularly in the case of the search and design of antifungal agents directed against the cell wall.

## Figures and Tables

**Table 1:** List of genes selected from the screening for Calcofluor white (CW) hypersensitivity, ordered according to their Gene Ontology annotations. CW sensitivity indicates the level of growth affectation in the presence of CW, from 0.0 (maximum effect) to 1.0 (absence of effect).

Gene	CW Sensitivity	Biological Process	Gene	CW Sensitivity	Biological Process
*CDC20*	0.10	Cell cycle	*CFT1*	0.14	mRNA processing
*CKS1*	0.09	Cell cycle	*DCP2*	0.14	mRNA processing
*GUK1*	0.16	Cell wall	*PRP31*	0.16	mRNA processing
*ARP7*	0.19	Chromatin organization	*NUP145*	0.18	Nuclear transport
*RSC58*	0.20	Chromatin organization	*PRE5*	0.18	Protein degradation
*RSC6*	0.08	Chromatin organization	*PRE6*	0.18	Protein degradation
*RSC8*	0.20	Chromatin organization	*RPT6*	0.17	Protein degradation
*SPT16*	0.20	Chromatin organization	*OST2*	0.07	Protein glycosylation
*STH1*	0.12	Chromatin organization	*RFT1*	0.10	Protein glycosylation
*TAF10*	0.15	Chromatin organization	*ROT1*	0.11	Protein glycosylation
*TAF5*	0.20	Chromatin organization	*SWP1*	0.18	Protein glycosylation
*ARC40*	0.18	Cytoskeleton organization	*VRG4*	0.06	Protein glycosylation
*PFY1*	0.06	Cytoskeleton organization	*SRP68*	0.19	Protein targeting
*MCM7*	0.18	DNA replication	*SRP72*	0.13	Protein targeting
*SLD5*	0.17	DNA replication	*YPP1*	0.17	Protein targeting
*BET3*	0.13	Golgi vesicle transport	*BRX1*	0.15	rRNA processing
*DOP1*	0.15	Golgi vesicle transport	*DBP9*	0.20	rRNA processing
*SEC14*	0.14	Golgi vesicle transport	*EBP2*	0.17	rRNA processing
*SEC18*	0.12	Golgi vesicle transport	*FAP7*	0.04	rRNA processing
*SEC2*	0.11	Golgi vesicle transport	*FCF1*	0.17	rRNA processing
*SFT1*	0.09	Golgi vesicle transport	*SPB1*	0.19	rRNA processing
*TIP20*	0.14	Golgi vesicle transport	*NOP2*	0.15	rRNA processing
*YIP1*	0.16	Golgi vesicle transport	*NOP15*	0.19	rRNA processing
*GAB1*	0.12	GPI biosynthesis	*MOT1*	0.18	Transcription
*MCD4*	0.18	GPI biosynthesis	*RPL18A*	0.17	Translation
*PHS1*	0.10	Lipid metabolism	*TIF35*	0.14	Translation

**Table 2:** List of genes corresponding to those mutants showing increased Mlp1-GFP levels under non-stress conditions. Mlp1-GFP ratio refers to the MFI (mean fluorescence intensity) value for each mutant respect to the wild-type. CW sensitivity denotes the sensitivity level to CW obtained from the phenotypic screen previously described. Those mutants with ratio ≤ 0.2 are highlighted in red and those with ratio ≤ 0.4 in orange. N.M.: non-measurable. References including previously described cell wall associations are listed.

Gene	Mlp1-GFP Ratio	CW Sensitivity	Biological Process	Cell Wall Association
*PGI1*	5.23	0.22	Carbohydrate metabolism	
*CDC12*	5.29	0.30	Cell cycle	
*MET30*	4.84	0.93	Cell cycle	
*MTW1*	4.65	N.M.	Chromosome segregation	
*SPC34*	7.09	1.41	Chromosome segregation	
*ARP2*	7.63	1.48	Cytoskeleton organization	
*COF1*	7.17	0.73	Cytoskeleton organization	[60]
*ARC35*	6.29	0.37	Cytoskeleton organization	
*LAS17*	3.57	0.87	Cytoskeleton organization	[61,62]
*TRS20*	3.01	0.38	Golgi vesicle transport	[63]
*SEC5*	3.95	0.44	Golgi vesicle transport	
*SEC3*	5.07	0.24	Golgi vesicle transport	[64]
*SEC4*	4.94	0.27	Golgi vesicle transport	[65]
*YPT1*	3.78	0.41	Golgi vesicle transport	[66]
*SEC15*	6.93	N.M.	Golgi vesicle transport	
*SEC24*	3.64	0.32	Golgi vesicle transport	
*SEC10*	9.46	0.50	Golgi vesicle transport	
*SAR1*	3.27	N.M.	Golgi vesicle transport	
*GPI17*	3.44	0.21	GPI biosynthesis	
*GPI16*	6.27	0.51	GPI biosynthesis	
*GAB1*	6.47	0.12	GPI biosynthesis	
*GPI12*	3.03	0.39	GPI biosynthesis	
*PGA1*	12.99	N.M.	GPI biosynthesis	
*RER2*	3.28	0.27	Lipid metabolism	[67]
*ERO1*	4.62	N.M.	Protein folding	
*ROT1*	7.97	0.11	Protein folding	[68,69]
*RFT1*	4.39	0.10	Protein glycosylation	
*ALG14*	5.72	0.23	Protein glycosylation	
*WBP1*	5.95	0.42	Protein glycosylation	
*PMI40*	3.04	0.91	Protein glycosylation	
*SEC53*	3.14	0.44	Protein glycosylation	
*VRG4*	2.96	0.06	Protein glycosylation	
*SWP1*	3.36	0.18	Protein glycosylation	
*ALG11*	3.07	0.21	Protein glycosylation	[70]
*OST2*	5.74	0.07	Protein glycosylation	
*DPM1*	3.34	0.46	Protein glycosylation	[67]
*SEC63*	4.22	0.82	Protein targeting	
*CDC42*	9.02	2.19	Signaling	[71,72]
*RPA190*	3.60	0.33	Transcription from RNA pol I	
*MOT1*	3.82	0.18	Transcription from RNA pol II	
*YNL171C*	5.45	1.23	Unknown	

## Data Availability

The data presented in this study are available on request from the corresponding author.

## Supplementary Materials

The following supporting information can be downloaded at: https://www.mdpi.com/article/10.3390/jof8070718/s1, Table S1: Calcofluor white hypersensitivity screening dataset; Table S2: Positive hits from the Calcofluor white hypersensitivity screening; Table S3: Mlp1-GFP expression screening dataset; Table S4: Positive hits from the Mlp1-GFP expression screening; Table S5: Mutant strains showing increased basal levels of Mlp1-GFP; Figure S1: CW susceptibility assays of some selected hits from the CW sensitivity screening. Figure S2: Fluorescence microscopy images of strains expressing Mlp1-GFP.
