# Supplementary material for: Systematic Identification of Essential Genes Required for Yeast Cell Wall Integrity: Involvement of the RSC Remodelling Complex
Source: J Fungi (Basel). 2022 Jul 8;8(7):718. doi: 10.3390/jof8070718 (PMC9323250; doi:10.3390/jof8070718)
Supplement: Supplementary file 1 [file jof-08-00718-s001.zip › Supplemental Figure S1.pdf]

Figure S1

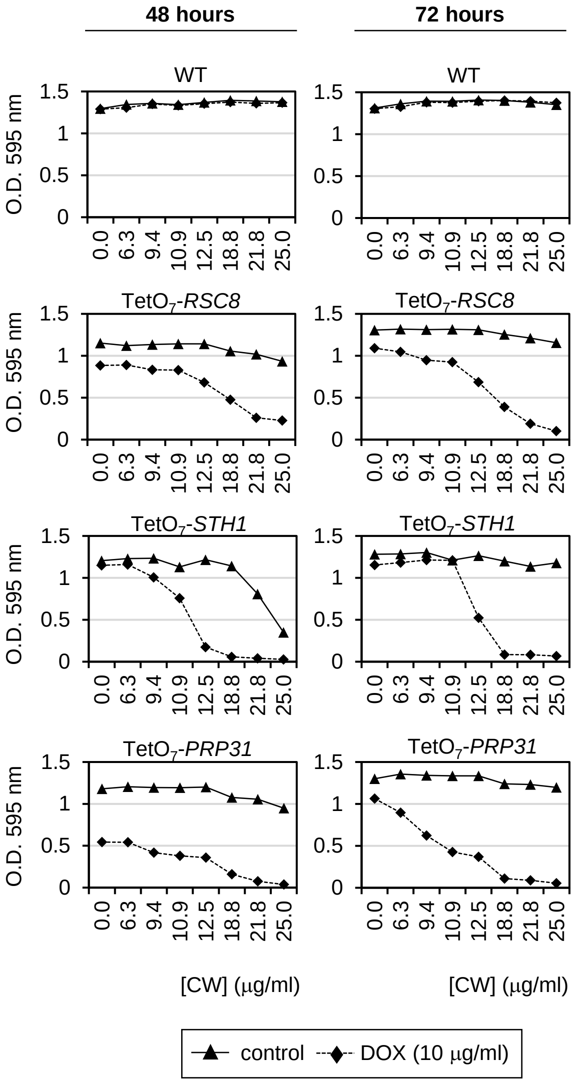

Figure S1: CW susceptibility assays of some selected hits from the CW sensitivity screening. Cell growth assays were carried out in 96-well microtiter plates, preparing serial dilutions of CW in a final volume of 150 µL of YPD including or not a constant concentration of doxycycline (10 µg/mL). Each well was inoculated with approximately 10^4 cells from a culture grown in YPD. Plates were incubated at 30ºC and cell growth was determined after 48 and 72 h by measuring the absorbance at 595 nm using a microplate reader.
