# Supplementary material for: Systematic Identification of Essential Genes Required for Yeast Cell Wall Integrity: Involvement of the RSC Remodelling Complex
Source: J Fungi (Basel). 2022 Jul 8;8(7):718. doi: 10.3390/jof8070718 (PMC9323250; doi:10.3390/jof8070718)
Supplement: Supplementary file 1 [file jof-08-00718-s001.zip › Supplemental Figure S2.pdf]

Figure S2

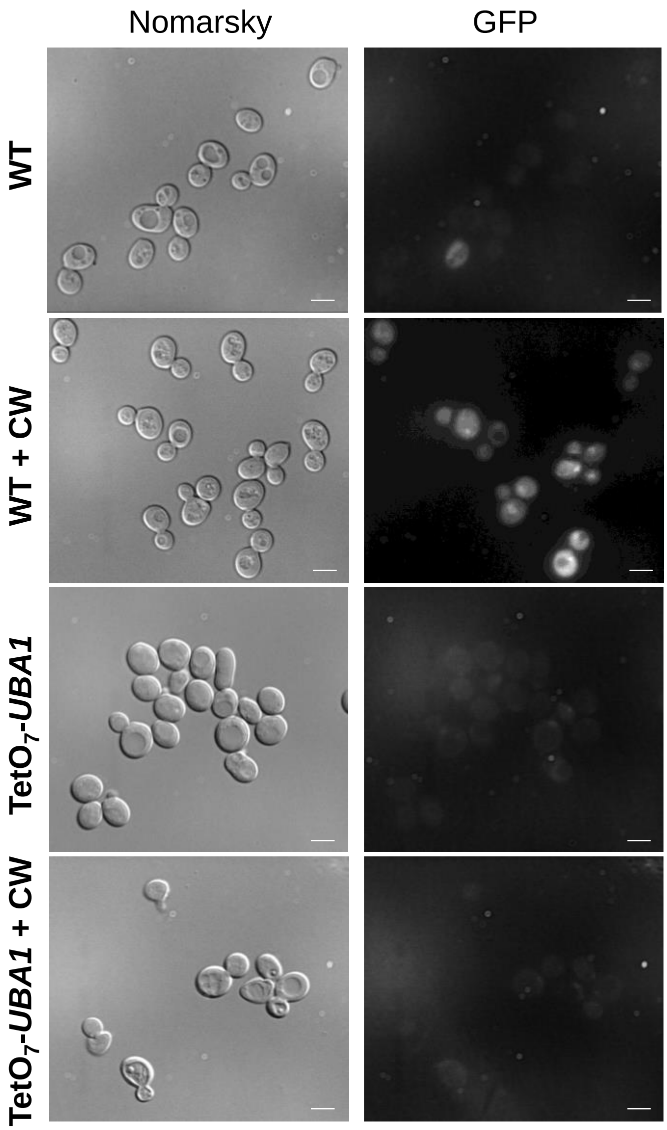

Figure S2: Fluorescence microscopy images of the wild-type (WT) and the TetO$_7$-*UBA1* strains transformed with the plasmid bearing the Mlp1-GFP construction grown in YPD plus doxycycline (10 µg/mL) and treated or not with CW (3h, 10 µg/mL). Scale bars: 5 µm.
