# Supplementary material for: Systematic Identification of Essential Genes Required for Yeast Cell Wall Integrity: Involvement of the RSC Remodelling Complex
Source: J Fungi (Basel). 2022 Jul 8;8(7):718. doi: 10.3390/jof8070718 (PMC9323250; doi:10.3390/jof8070718)
Supplement: Supplementary file 1 [file jof-08-00718-s001.zip › Supplemental Table S1.pdf]

Table S1. Results from the Calcofluor white (CW) sensitivity screening using the complete Tet-promoters Hughes collection (yTHC). Sensitivit were calculated as described in Materials and Methods. N.M.: Non-Measurable.

Mutated ORF	Mutated gene	CW ratio
YAL003W	EFB1	1.34
YAL025C	MAK16	0.42
YAL033W	POP5	1.03
YAL034W-A	MTW1	N.M.
YAL038W	CDC19	0.27
YAL043C	PTA1	1.29
YAR008W	SEN34	0.31
YAR019C	CDC15	1.00
YBL004W	UTP20	0.54
YBL014C	RRN6	0.96
YBL018C	POP8	1.05
YBL020W	RFT1	0.15
YBL030C	PET9	N.M.
YBL034C	STU1	1.20
YBL035C	POL12	0.95
YBL040C	ERD2	0.20
YBL050W	SEC17	0.25
YBL073W	YBL073W	1.01
YBL074C	AAR2	0.99
YBL076C	ILS1	0.46
YBL077W	YBL077W	1.01
YBL084C	CDC27	1.08
YBR002C	RER2	0.27
YBR011C	IPP1	0.34
YBR029C	CDS1	0.11
YBR049C	REB1	0.94
YBR055C	PRP6	0.91
YBR060C	ORC2	0.97
YBR070C	ALG14	0.23
YBR079C	RPG1	0.47
YBR080C	SEC18	0.15
YBR086C	IST2	0.96
YBR087W	RFC5	0.97
YBR088C	POL30	0.28
YBR102C	EXO84	1.23
YBR110W	ALG1	0.95
YBR135W	CKS1	0.16
YBR136W	MEC1	1.01
YBR140C	IRA1	0.95
YBR142W	MAK5	1.10
YBR143C	SUP45	1.00
YBR153W	RIB7	1.00
YBR155W	CNS1	0.96
YBR167C	POP7	0.98
YBR168W	YBR168W	0.99
YBR170C	NPL4	0.97
YBR190W	YBR190W	0.72
YBR192W	RIM2	1.01
YBR193C	MED8	1.26
YBR196C	PGI1	0.19
YBR198C	TAF5	0.13
YBR202W	MCM7	0.12
YBR211C	AME1	1.03
YBR234C	ARC40	0.19
YBR236C	ABD1	1.00
YBR237W	PRP5	0.96
YBR243C	ALG7	0.36
YBR252W	DUT1	1.09
YBR253W	SRB6	0.97
YBR254C	TRS20	0.38
YBR256C	RIB5	1.05
YBR257W	POP4	1.04
YCL004W	PGS1	1.06
YCL017C	NFS1	1.05
YCL053C	YCL053C	0.99
YCL054W	SPB1	0.17
YCL059C	KRR1	1.13
YCR013C	YCR013C	0.99
YCR035C	RRP43	0.39
YCR042C	TAF2	1.08
YCR052W	RSC6	0.14
YCR072C	YCR072C	1.01
YDL007W	RPT2	0.33
YDL008W	APC11	1.05
YDL015C	TSC13	0.56
YDL016C	YDL016C	0.94
YDL028C	MPS1	0.99
YDL029W	ARP2	1.48
YDL030W	PRP9	0.94
YDL031W	DBP10	0.27
YDL043C	PRP11	0.96
YDL045C	FAD1	0.99
YDL055C	PSA1	0.13
YDL058W	USO1	1.01
YDL060W	TSR1	0.89
YDL064W	UBC9	1.09
YDL087C	LUC7	1.06
YDL092W	SRP14	0.06
YDL098C	SNU23	0.90
YDL102W	CDC2	0.98
YDL103C	QRI1	0.99
YDL105W	QRI2	0.95
YDL108W	KIN28	0.98
YDL111C	RRP42	0.96
YDL126c	CDC48	0.52
YDL132W	CDC53	1.05
YDL139C	SCM3	0.94
YDL140C	RPO21	0.53
YDL141W	BPL1	0.99
YDL143W	CCT4	1.13
YDL145C	COP1	0.96
YDL147W	RPN5	0.41
YDL148C	NOP14	1.02
YDL150W	RPC53	1.08
YDL153C	SAS10	1.29
YDL164c	CDC9	0.98
YDL166C	FAP7	0.01
YDL193W	NUS1	0.89

Mutated ORF	Mutated gene	CW ratio	Mutated ORF	Mutated gene	CW ratio	Mutated ORF	Mutated gene	CW ratio
YDL195W	SEC31	0.25	YDR228C	PCF11	1.10	YDR454C	GUK1	0.19
YDL196W	YDL196W	1.00	YDR232W	HEM1	0.32	YDR460W	TFB3	1.22
YDL207W	GLE1	0.80	YDR235W	PRP42	1.05	YDR464W	SPP41	0.38
YDL209C	CWC2	1.07	YDR236C	FMN1	1.08	YDR472W	TRS31	0.91
YDL217C	TIM22	1.17	YDR238C	SEC26	0.81	YDR473C	PRP3	0.96
YDL220C	CDC13	0.99	YDR240C	SNU56	1.06	YDR478W	SNM1	1.00
YDR013W	YDR013W	1.07	YDR243C	PRP28	1.07	YDR489W	SLD5	0.17
YDR016C	DAD1	0.55	YDR246W	TRS23	1.01	YDR498C	SEC20	1.11
YDR021W	FAL1	1.02	YDR267C	YDR267C	1.05	YDR527W	RBA50	0.98
YDR023W	SES1	0.15	YDR280W	RRP45	0.14	YDR531W	YDR531W	0.94
YDR037W	KRS1	0.48	YDR288W	YDR288W	1.09	YEL002C	WBP1	0.42
YDR044W	HEM13	1.05	YDR292C	SRP101	0.93	YEL019C	MMS21	1.02
YDR045C	RPC11	0.90	YDR299W	BFR2	0.35	YEL032W	MCM3	1.02
YDR047W	HEM12	0.48	YDR301W	CFT1	0.18	YEL034W	HYP2	0.30
YDR050C	TPI1	N.M.	YDR302W	GPI11	1.02	YEL055C	POL5	1.01
YDR052C	DBF4	0.93	YDR308C	SRB7	1.04	YEL058W	PCM1	1.00
YDR054C	CDC34	0.87	YDR311W	TFB1	0.87	YER003C	PMI40	0.91
YDR060W	MAK21	0.35	YDR324c	YDR324C	0.95	YER006W	NUG1	0.21
YDR062W	LCB2	1.08	YDR325W	YCG1	1.04	YER008C	SEC3	0.24
YDR087C	RRP1	0.34	YDR327W	YDR327W	0.98	YER009W	NTF2	0.99
YDR088C	SLU7	0.97	YDR339C	FCF1	0.15	YER012W	PRE1	1.00
YDR091C	RLI1	0.23	YDR341C	YDR341C	0.26	YER013W	PRP22	1.01
YDR113C	PDS1	1.27	YDR353W	TRR1	1.02	YER022W	SRB4	1.05
YDR118W	APC4	0.99	YDR356W	NUF1	N.M.	YER023W	PRO3	0.95
YDR141C	DOP1	0.16	YDR361C	BCP1	0.67	YER026C	CHO1	1.06
YDR145W	TAF12	1.06	YDR365c	ESF1	0.27	YER029C	SMB1	0.50
YDR160W	SSY1	1.01	YDR367W	KEI1	0.57	YER043C	SAH1	0.82
YDR164C	SEC1	1.20	YDR373W	FRQ1	1.66	YER048C	CAJ1	1.01
YDR166C	SEC5	1.10	YDR376W	ARH1	1.11	YER082C	UTP7	0.53
YDR167W	TAF10	0.16	YDR396W	YDR396W	1.05	YER093C	TSC11	1.04
YDR168w	CDC37	1.00	YDR397C	NCB2	0.90	YER112W	LSM4	1.10
YDR177W	UBC1	0.98	YDR398W	UTP5	0.71	YER125W	RSP5	0.74
YDR182W	CDC1	1.01	YDR407C	TRS120	0.96	YER127W	LCP5	1.52
YDR188W	CCT6	1.09	YDR412W	YDR412W	0.45	YER146W	LSM5	0.95
YDR189W	SLY1	0.34	YDR413C	YDR413C	1.14	YER165W	PAB1	0.70
YDR190C	RVB1	0.90	YDR416W	SYF1	1.01	YER168C	CCA1	1.00
YDR196C	YDR196C	1.01	YDR429C	TIF35	0.18	YER171W	RAD3	0.33
YDR201W	SPC19	1.01	YDR434W	GPI17	0.21	YER172C	BRR2	0.93
YDR208W	MSS4	1.08	YDR437W	YDR437W	1.02	YFL002C	SPB4	1.03
YDR211W	STN1	1.21	YDR449c	YDR449C	0.56	YFL005W	SEC4	0.27

Mutated ORF	Mutated gene	CW ratio	Mutated ORF	Mutated gene	CW ratio	Mutated ORF	Mutated gene	CW ratio
YFL008w	SMC1	1.01	YGL130W	CEG1	1.14	YGR175C	ERG1	0.11
YFL009W	CDC4	0.47	YGL137W	SEC27	0.40	YGR179C	OKP1	1.05
YFL017C	GNA1	1.04	YGL142C	GPI10	1.02	YGR185C	TYS1	0.99
YFL018W-A	LPD1	0.29	YGL145W	TIP20	0.16	YGR186W	TFG1	1.00
YFL024C	EPL1	0.68	YGL155W	CDC43	0.96	YGR190C	YGR190C	1.14
YFL029C	CAK1	1.00	YGL171W	ROK1	0.98	YGR191W	HIP1	1.06
YFL035C	MOB2	1.09	YGL172W	NUP49	0.85	YGR195W	SKI6	0.86
YFL038C	YPT1	0.41	YGL207W	SPT16	0.15	YGR198w	YPP1	0.15
YFL045C	SEC53	0.44	YGL225W	VRG4	0.09	YGR211W	ZPR1	0.91
YFR002W	NIC96	0.50	YGL233W	SEC15	N.M.	YGR216C	GPI1	0.96
YFR003C	YFR003C	1.05	YGL238W	CSE1	1.33	YGR218W	CRM1	0.23
YFR004W	RPN11	0.22	YGL245W	GUS1	0.89	YGR245C	SDA1	0.22
YFR005C	SAD1	0.98	YGL247W	BRR6	0.97	YGR251w	NOP19	0.83
YFR028c	CDC14	1.05	YGR002C	GOD1	1.00	YGR255C	COQ6	1.03
YFR031C	SMC2	0.95	YGR005C	TFG2	1.19	YGR264C	MES1	1.04
YFR037C	RSC8	0.20	YGR013W	SNU71	0.98	YGR267C	FOL2	0.84
YFR050C	PRE4	1.02	YGR030C	POP6	0.26	YGR274C	TAF1	0.74
YFR051C	RET2	0.87	YGR046W	YGR046W	1.15	YGR277C	YGR277C	0.96
YGL001C	ERG26	N.M.	YGR047C	TFC4	0.97	YGR278W	CWC22	1.02
YGL008C	PMA1	0.33	YGR048W	UFD1	0.39	YGR280c	YGR280C	0.98
YGL018C	JAC1	1.00	YGR060W	ERG25	0.51	YGR283c	YGR283C	0.90
YGL044C	RNA15	0.98	YGR065C	VHT1	1.00	YHR005C	GPA1	1.02
YGL047W	YGL047W	1.07	YGR074W	SMD1	0.13	YHR019C	DED81	0.52
YGL048C	RPT6	0.18	YGR075C	PRP38	0.75	YHR023W	MYO1	1.14
YGL061C	DUO1	1.08	YGR083C	GCD2	1.11	YHR036W	YHR036W	1.00
YGL065C	ALG2	1.66	YGR090w	UTP22	0.21	YHR040W	YHR040W	0.39
YGL068W	USE1	1.13	YGR091W	PRP31	0.09	YHR042W	NCP1	1.04
YGL069C	SRF3	1.02	YGR094W	VAS1	0.25	YHR058C	MED6	0.21
YGL073W	HSF1	0.96	YGR095C	RRP46	0.25	YHR062C	RPP1	1.07
YGL075C	MPS2	1.09	YGR098C	ESP1	1.00	YHR068W	DYS1	1.10
YGL091C	NBP35	1.10	YGR099W	TEL2	0.94	YHR069C	RRP4	0.30
YGL092W	NUP145	0.11	YGR103w	NOP7	0.18	YHR070W	TRM5	1.08
YGL093W	SPC105	0.99	YGR116W	SPT6	0.50	YHR072W	ERG7	0.89
YGL098W	YGL098W	1.15	YGR119C	NUP57	1.00	YHR072W-A	NOP10	1.00
YGL103W	RPL28	0.06	YGR120C	SEC35	1.03	YHR074W	QNS1	1.01
YGL106W	MLC1	1.09	YGR128C	UTP8	1.07	YHR083W	YHR083W	1.12
YGL108C	YGL108C	0.95	YGR147C	NAT2	1.01	YHR085W	IPI1	1.00
YGL112C	TAF6	2.30	YGR158C	MTR3	0.88	YHR088W	RPF1	0.96
YGL116W	CDC20	0.10	YGR172C	YIP1	0.20	YHR089C	GAR1	0.99
YGL122C	NAB2	2.02	YGR173w	GIR1	1.07	YHR090C	YNG2	0.75

Mutated ORF	Mutated gene	CW ratio	Mutated ORF	Mutated gene	CW ratio	Mutated ORF	Mutated gene	CW ratio
YHR099W	TRA1	0.85	YIR022W	SEC11	1.18	YKL012W	PRP40	1.04
YHR101C	BIG1	0.88	YJL001W	PRE3	0.96	YKL014C	YKL014C	1.17
YHR102W	KIC1	1.04	YJL009W	YJL009W	0.99	YKL018W	SWD2	1.07
YHR107C	CDC12	0.30	YJL011C	RPC17	0.94	YKL021C	MAK11	1.06
YHR118C	ORC6	0.93	YJL033W	HCA4	2.04	YKL022C	CDC16	1.07
YHR122W	YHR122W	0.21	YJL039C	NUP192	0.34	YKL024C	URA6	0.49
YHR143W-A	RPC10	1.08	YJL042W	MHP1	1.11	YKL033W	YKL033W	1.03
YHR164C	DNA2	0.35	YJL050W	MTR4	0.17	YKL035W	UGP1	0.39
YHR165C	PRP8	1.04	YJL061W	NUP82	N.M.	YKL045W	PRI2	0.91
YHR166C	CDC23	0.27	YJL069C	UTP18	0.52	YKL052C	ASK1	N.M.
YHR169W	DBP8	1.07	YJL072C	YJL072C	1.03	YKL059C	YKL059C	1.10
YHR170W	NMD3	0.43	YJL074C	SMC3	1.00	YKL078W	JA2	1.21
YHR172W	SPC97	1.03	YJL076W	NET1	N.M.	YKL082C	RRP14	0.99
YHR174W	ENO2	1.05	YJL081C	ARP4	0.77	YKL083w	YKL083W	1.00
YHR186C	KOG1	0.99	YJL090C	DPB11	0.80	YKL088W	YKL088W	0.97
YHR188C	GPI16	0.51	YJL091C	YJL091C	0.97	YKL089W	MIF2	1.02
YHR196w	UTP9	0.93	YJL097W	PHS1	0.11	YKL095W	YJU2	1.04
YHR197W	IPI2	1.01	YJL125C	GCD14	0.98	YKL099C	UTP11	0.26
YHR205W	SCH9	0.94	YJL156C	SSY5	0.99	YKL108W	SLD2	0.87
YIL003W	DRE3	0.26	YJL194W	CDC6	N.M.	YKL111C	YKL111C	1.06
YIL004C	BET1	0.21	YJL202C	YJL202C	0.93	YKL112W	ABF1	N.M.
YIL021W	RPB3	0.97	YJL203W	PRP21	0.97	YKL122C	SRP21	1.11
YIL026C	IRR1	1.37	YJR002W	MPP10	0.65	YKL125W	RRN3	1.18
YIL046W	MET30	0.93	YJR007W	SUI2	1.18	YKL139W	CTK1	1.03
YIL048W	NEO1	0.80	YJR017C	ESS1	1.05	YKL141W	SDH3	0.98
YIL061C	SNP1	0.98	YJR022W	LSM8	1.05	YKL144C	RPC25	0.70
YIL078w	THS1	0.21	YJR041C	YJR041C	1.01	YKL153W	YKL153W	1.02
YIL106W	MOB1	1.02	YJR042W	NUP85	1.18	YKL154W	SRP102	1.14
YIL109C	SEC24	0.32	YJR046W	YJR046W	0.99	YKL165C	MCD4	0.16
YIL115C	NUP159	1.00	YJR057W	CDC8	0.99	YKL172W	EBP2	0.15
YIL126W	STH1	0.08	YJR058C	APS2	0.86	YKL180W	RPL17A	0.11
YIL144W	TID3	0.96	YJR067C	YAE1	1.05	YKL186C	MTR2	0.38
YIL147C	SLN1	1.11	YJR068W	RFC2	0.97	YKL189W	HYM1	0.98
YIL150C	MCM10	0.93	YJR072C	YJR072C	0.31	YKL193C	SDS22	0.97
YIR006C	PAN1	1.06	YJR076C	CDC11	0.99	YKL196C	YKT6	1.02
YIR008C	PRI1	1.10	YJR093C	FIP1	1.04	YKL203C	TOR2	1.02
YIR010W	YIR010W	0.22	YJR123W	RPS5	1.95	YKL210w	UBA1	0.20
YIR011c	STS1	1.02	YJR141W	YJR141W	0.94	YKR002W	PAP1	1.43
YIR012W	SQT1	0.17	YKL006C-A	SFT1	0.08	YKR008W	RSC4	0.36
YIR015W	RPR2	1.03	YKL009W	MRT4	1.06	YKR022C	YKR022C	1.04

Mutated ORF	Mutated gene	CW ratio
YKR025W	RPC37	3.14
YKR037C	SPC34	1.41
YKR062W	TFA2	1.02
YKR063C	LAS1	1.03
YKR068C	BET3	0.08
YKR071C	YKR071C	0.94
YKR079C	YKR079C	1.03
YKR083C	DAD2	0.93
YKR086W	PRP16	N.M.
YLL003W	SFI1	0.67
YLL004W	ORC3	1.03
YLL008W	DRS1	0.80
YLL018C	DPS1	0.64
YLL034c	YLL034c	0.96
YLL035W	GRC3	0.63
YLL036C	PRP19	0.39
YLL037W	YLL037W	1.06
YLL050C	COF1	0.02
YLR002c	NOC3	0.90
YLR005W	SSL1	1.03
YLR007W	NSE1	1.10
YLR008C	YLR008C	0.64
YLR009W	RLP24	0.17
YLR010C	TEN1	0.98
YLR022C	YLR022C	1.00
YLR026C	SED5	0.38
YLR029C	RPL15A	0.14
YLR033W	RSC58	0.10
YLR045C	STU2	1.01
YLR060W	FRS1	0.43
YLR066W	SPC3	0.96
YLR071C	RGR1	0.97
YLR076C	YLR076C	0.97
YLR078C	BOS1	1.03
YLR086W	SMC4	0.57
YLR088W	GAA1	1.03
YLR100W	ERG27	1.02
YLR101C	YLR101C	1.04
YLR103C	CDC45	0.77
YLR105C	SEN2	0.91

Mutated ORF	Mutated gene	CW ratio
YLR106C	MDN1	0.97
YLR115W	CFT2	1.07
YLR117C	CLF1	0.99
YLR127C	APC2	0.98
YLR129W	DIP2	1.01
YLR132C	YLR132C	0.99
YLR140W	YLR140W	1.04
YLR141W	RRN5	0.79
YLR145W	YLR145W	0.73
YLR147C	SMD3	0.63
YLR153C	ACS2	2.05
YLR163C	MAS1	0.82
YLR166C	SEC10	0.07
YLR167W	RPS31	1.10
YLR175W	CBF5	0.47
YLR186W	EMG1	0.57
YLR195C	NMT1	0.98
YLR196w	PWP1	0.95
YLR198C	YLR198C	0.91
YLR208W	SEC13	0.94
YLR212c	TUB4	1.04
YLR223C	IFH1	0.92
YLR229C	CDC42	2.19
YLR243W	YLR243W	1.01
YLR249W	YEF3	1.09
YLR259C	YLR259C	0.98
YLR272C	LOC7	1.06
YLR274W	CDC46	1.02
YLR275W	SMD2	1.15
YLR276C	DBP9	0.16
YLR277C	YSH1	0.89
YLR291C	GCD7	0.67
YLR298C	YHC1	0.95
YLR305C	STT4	0.22
YLR310C	CDC25	1.08
YLR314C	CDC3	1.06
YLR316c	TAD3	0.95
YLR323C	CWC24	1.15
YLR347C	KAP95	1.51
YLR355C	ILV5	1.13

Mutated ORF	Mutated gene	CW ratio
YLR359W	ADE13	0.48
YLR378C	SEC61	1.23
YLR383W	RHC18	1.04
YLR397C	AFG2	0.09
YLR419w	YLR419W	0.98
YLR424W	YLR424W	1.06
YLR430W	SEN1	0.98
YLR438W	CAR2	1.05
YLR440C	SEC39	0.08
YLR457C	NBP1	0.93
YLR459W	GAB1	0.19
YML015C	TAF11	0.30
YML023C	YML023C	0.87
YML025C	YML025C	1.15
YML031W	NDC1	0.08
YML043C	YML043C	1.05
YML049C	RSE1	1.02
YML064C	YML064C	1.05
YML065W	YML065W	0.97
YML069W	POB3	1.25
YML077W	BET5	0.98
YML085C	YML085C	1.59
YML091c	RPM2	0.98
YML092C	PRE8	0.99
YML093W	UTP14	1.06
YML098W	TAF13	1.12
YML105C	SEC65	1.01
YML114C	TAF65	1.06
YML125c	YML125C	0.95
YML126C	ERG13	0.64
YML127W	RSC9	0.23
YML130C	ERO1	N.M.
YMR001C	CDC5	1.02
YMR005W	TAF4	0.60
YMR013C	SEC59	0.96
YMR028W	TAP42	1.00
YMR033W	ARP9	1.00
YMR043W	MCM1	0.23
YMR047C	NUP116	1.08
YMR059W	SEN15	1.07

Mutated ORF	Mutated gene	CW ratio
YMR061W	RNA14	0.99
YMR076C	PDS5	0.69
YMR079W	SEC14	0.16
YMR093W	YMR093W	0.50
YMR094W	CTF13	1.06
YMR112C	MED11	0.96
YMR113W	FOL3	0.91
YMR117C	SPC24	1.06
YMR128W	ECM16	0.97
YMR134W	YMR134W	1.69
YMR146C	TIF34	0.50
YMR149W	SWP1	0.06
YMR186w	HSC82	0.98
YMR197C	YMR197C	1.21
YMR200W	ROT1	0.07
YMR203W	TOM40	0.96
YMR208W	ERG12	0.26
YMR211W	DML1	1.01
YMR213W	YMR213W	2.29
YMR218C	TRS130	0.98
YMR220W	ERG8	1.04
YMR227C	TAF7	0.97
YMR235C	RNA1	0.18
YMR236W	TAF9	0.14
YMR239C	RNT1	0.66
YMR240C	CUS1	1.46
YMR260C	TIF11	1.02
YMR268C	PRP24	1.20
YMR270C	RRN9	1.05
YMR281W	GPI12	0.39
YMR288W	HSH155	0.99
YMR290C	HAS1	0.41
YMR296C	LCB1	0.85
YMR308C	PSE1	0.86
YMR309C	NIP1	1.00
YMR314W	PRE5	0.15
YNL002C	RLP7	1.07
YNL006W	LST8	1.04
YNL007C	SIS1	1.04
YNL026W	SAM50	1.00

Mutated ORF	Mutated gene	CW ratio
YNL038W	YNL038W	0.83
YNL039W	TFC5	0.76
YNL048W	ALG11	0.21
YNL061W	NOP2	0.12
YNL062C	YNL062C	1.00
YNL088W	YNL088W	0.96
YNL102W	POL1	0.24
YNL110c	NOP15	0.18
YNL113W	RPC19	0.18
YNL118C	DCP2	0.16
YNL124W	NAF1	1.20
YNL126W	SPC98	0.46
YNL131W	TOM22	1.12
YNL149C	YNL149C	1.19
YNL150W	YNL150W	1.13
YNL151C	RPC31	0.42
YNL158W	PGA1	N.M.
YNL161W	CBK1	1.03
YNL163C	RIA1	0.52
YNL171C	YNL171C	1.23
YNL181W	YNL181W	1.27
YNL182c	YNL182C	0.30
YNL188W	KAR1	0.96
YNL207W	RIO2	0.26
YNL216W	RAP1	1.02
YNL221C	YNL221C	1.14
YNL222W	SSU72	0.72
YNL232W	CSL4	0.95
YNL244C	SUI1	0.18
YNL245C	YNL245C	0.58
YNL247W	YNL247W	1.00
YNL251C	NRD1	0.06
YNL256W	FOL1	0.96
YNL258C	DSL1	N.M.
YNL260C	YNL260C	0.80
YNL261W	ORC5	0.79
YNL262W	POL2	0.25
YNL263C	YIF1	1.03
YNL267w	PIK1	0.22
YNL272C	SEC2	0.16

Mutated ORF	Mutated gene	CW ratio
YNL282W	POP3	1.00
YNL287W	SEC21	1.04
YNL290W	RFC3	0.95
YNL308C	YNL308C	1.12
YNL310C	YNL310C	0.92
YNL312W	RFA2	1.01
YNL313c	YNL313C	0.39
YNL317W	PFS2	0.39
YNR026C	SEC12	0.99
YNR035C	ARC35	0.37
YNR038w	DBP6	1.01
YNR043W	MVD1	0.97
YNR046W	YNR046W	0.96
YNR053C	NOG2	0.19
YNR054C	YNR054C	0.99
YOL005C	RPB11	0.44
YOL010W	RCL1	0.98
YOL021C	DIS3	0.30
YOL022C	YOL022C	1.03
YOL026C	YOL026C	1.02
YOL034W	YOL034W	0.96
YOL038W	PRE6	0.17
YOL069W	NUF2	0.60
YOL077c	BRX1	0.18
YOL078W	YOL078W	1.05
YOL094C	RFC4	0.99
YOL097C	WRS1	0.87
YOL102C	TPT1	0.97
YOL120C	RPL18A	0.20
YOL130W	ALR1	0.87
YOL133W	HRT1	1.00
YOL135C	MED7	0.99
YOL139C	CDC33	0.21
YOL142W	RRP40	0.48
YOL144W	YOL144W	1.02
YOL149W	DCP1	N.M.
YOR004W	YOR004W	1.07
YOR048C	RAT1	0.30
YOR057W	SGT1	0.98
YOR060C	YOR060C	0.91

Mutated ORF	Mutated gene	CW ratio
YOR063W	RPL3	0.96
YOR074C	CDC21	1.06
YOR077w	RTS2	1.04
YOR095C	RKI1	0.90
YOR098C	NUP1	0.97
YOR102W	YOR102W	0.96
YOR103C	OST2	0.05
YOR110W	TFC7	0.98
YOR116C	RPO31	0.27
YOR119c	RIO1	0.32
YOR122C	PFY1	0.09
YOR143C	THI80	1.01
YOR145c	YOR145C	0.99
YOR146W	YOR146W	1.00
YOR148C	SPP2	0.97
YOR149C	SMP3	0.99
YOR151C	RPB2	0.21
YOR159C	SME1	1.05
YOR168W	GLN4	0.30
YOR169C	YOR169C	1.01
YOR174W	MED4	1.03
YOR176W	YOR176W	1.01
YOR181W	LAS17	0.87
YOR194C	TOA1	0.17
YOR204W	DED1	0.51
YOR206W	YOR206W	0.90
YOR207C	RET1	1.00
YOR210W	RPB10	0.19
YOR218C	YOR218C	1.04
YOR224C	RPB8	0.61
YOR232W	MGE1	1.49
YOR236W	DFR1	1.00
YOR244W	ESA1	1.16
YOR249C	APC5	0.40
YOR254C	SEC63	0.82
YOR257W	CDC31	1.02
YOR259C	RPT4	0.27
YOR261C	RPN8	0.23
YOR262W	YOR262W	0.82
YOR272W	YTM1	0.34

Mutated ORF	Mutated gene	CW ratio
YOR278W	YOR278W	0.99
YOR281C	PLP2	0.65
YOR287C	YOR287C	1.00
YOR294W	RRS1	0.27
YOR326W	MYO2	1.11
YOR335C	ALA1	0.21
YOR336W	YOR336W	1.06
YOR340C	RPA43	0.85
YOR341W	RPA190	0.33
YOR353C	SOG2	0.97
YOR361C	PRT1	1.06
YOR370C	MRS6	0.85
YOR372C	NDD1	0.23
YPL007C	TFC8	1.00
YPL010W	RET3	0.29
YPL011C	TAF3	0.78
YPL012W	RRP12	0.70
YPL016W	SWI1	0.99
YPL020C	ULP1	1.09
YPL028w	ERG10	0.47
YPL043W	NOP4	0.33
YPL063W	TIM50	1.19
YPL075W	GCR1	0.78
YPL076W	GPI2	0.91
YPL082C	MOT1	0.11
YPL083C	SEN54	1.04
YPL093W	NOG1	0.23
YPL124W	SPC29	N.M.
YPL126W	NAN1	1.13
YPL128C	TBF1	0.71
YPL142C	YPL142C	1.02
YPL151C	YPL151C	1.64
YPL169C	MEX67	0.19
YPL190C	NAB3	0.21
YPL204W	YPL204W	N.M.
YPL210C	SRP72	0.14
YPL217C	BMS1	0.25
YPL218W	SAR1	N.M.
YPL228W	CET1	0.93
YPL231W	FAS2	N.M.

Mutated ORF	Mutated gene	CW ratio
YPL233W	NSL1	1.05
YPL235W	RVB2	0.56
YPL242C	IQG1	0.32
YPL243W	SRP68	0.15
YPL252C	YAH1	1.00
YPL266W	DIM1	0.31
YPR016C	TIF6	0.20
YPR019W	CDC54	1.00
YPR033C	HTS1	0.89
YPR034W	ARP7	0.20
YPR048W	TAH18	1.01
YPR082C	DIB1	1.26
YPR085C	YPR085C	1.00
YPR086W	SUA7	1.01
YPR094W	RDS3	0.99
YPR105C	COG4	N.M.
YPR107C	YTH1	1.13
YPR110C	RPC40	1.06
YPR112C	YPR112C	1.19
YPR133C	IWS1	1.09
YPR137W	RRP9	0.92
YPR144C	NOC4	0.22
YPR161C	YPR161C	1.03
YPR162C	ORC4	1.00
YPR168W	NUT2	0.96
YPR169W	YPR169W	1.29
YPR175W	DPB2	0.93
YPR178W	PRP4	0.27
YPR180W	AOS1	2.27
YPR183W	DPM1	0.46
YPR186C	PZF1	1.00
YPR187W	RPO26	0.40
YPR190C	RPC82	0.96

:y levels
