# Supplementary material for: Systematic Identification of Essential Genes Required for Yeast Cell Wall Integrity: Involvement of the RSC Remodelling Complex
Source: J Fungi (Basel). 2022 Jul 8;8(7):718. doi: 10.3390/jof8070718 (PMC9323250; doi:10.3390/jof8070718)
Supplement: Supplementary file 1 [file jof-08-00718-s001.zip › Supplemental Table S2.pdf]

Table S2. Positive hits from the Calcofluor white (CW) hypersensitivity screening. For each mutant strain data from three independent experiments (exp) and their corresponding mean value are shown.

Mutated ORF	Mutated gene	CW sensitivity (exp 1)	CW sensitivity (exp 2)	CW sensitivity (exp 3)	Mean	Biological process	Description from the *Saccharomyces Genome Database*
YBL020W	*RFT1*	0.15	0.07	0.07	**0.10**	Protein glycosylation	Membrane protein required for translocation of Man5GlcNac2-PP-Dol; required for translocation of Man5GlcNac2-PP-Dol from the cytoplasmic side to the lumenal side of the ER membrane but is not the flippase; mutation is suppressed by expression of human p53 protein; essential gene
YBR080C	*SEC18*	0.15	0.11	0.09	**0.12**	Golgi vesicle transport	AAA ATPase and SNARE disassembly chaperone; required for vesicular transport between ER and Golgi, the 'priming' step in homotypic vacuole fusion, autophagy, and protein secretion; releases Sec17p from SNAP complexes; has similarity to mammalian N-ethylmaleimide-sensitive factor (NSF)
YBR135W	*CKS1*	0.16	0.10	0.03	**0.09**	Cell cycle	Cyclin-dependent protein kinase regulatory subunit and adaptor; interacts with Cdc28p (aka Cdk1p); required for G1/S and G2/M phase transitions and budding; mediates phosphorylation and degradation of Sic1p; modulates proteolysis of M-phase targets through interactions with the proteasome; role in transcriptional regulation, recruiting proteasomal subunits to target gene promoters; human homologs CKS1B and CKS2 can each complement yeast cks1 null mutant
YBR198C	*TAF5*	0.13	0.34	0.15	**0.20**	Chromatin organization	Subunit (90 kDa) of TFIID and SAGA complexes; involved in RNA polymerase II transcription initiation and in chromatin modification
YBR202W	*MCM7*	0.12	0.36	0.07	**0.18**	DNA replication	Component of the Mcm2-7 hexameric helicase complex; MCM2-7 primes origins of DNA replication in G1 and becomes an active ATP-dependent helicase that promotes DNA melting and elongation in S-phase; forms an Mcm4p-6p-7p subcomplex
YBR234C	*ARC40*	0.19	0.26	0.08	**0.18**	Cytoskeleton organization	Subunit of the ARP2/3 complex; ARP2/3 is required for the motility and integrity of cortical actin patches
YCL054W	*SPB1*	0.17	0.18	0.21	**0.19**	rRNA processing	AdoMet-dependent methyltransferase; involved in rRNA processing and 60S ribosomal subunit maturation; methylates G2922 in the tRNA docking site of the large subunit rRNA and in the absence of snR52, U2921; suppressor of PAB1 mutants
YCR052W	*RSC6*	0.14	0.07	0.04	**0.08**	Chromatin organization	Component of the RSC chromatin remodeling complex; essential for mitotic growth; RSC6 has a paralog, SNF12, that arose from the whole genome duplication

YDL166C	*FAP7*	0.01	0.06	0.04	**0.04**	rRNA processing	Essential NTPase required for small ribosome subunit synthesis; mediates processing of the 20S pre-rRNA at site D in the cytoplasm but associates only transiently with 43S preribosomes via Rps14p; complex with Rps14 is conserved between humans, yeast, and arches; may be the endonuclease for site D; depletion leads to accumulation of pre-40S ribosomes in 80S-like ribosomes; human TAF9 functionally complements the lethality of the null mutation
YDR141C	*DOP1*	0.16	0.20	0.08	**0.15**	Golgi vesicle transport	Protein involved in vesicular transport at trans-Golgi network (TGN); TGN-localized, leucine-zipper domain protein; involved in endosome-to-Golgi transport during endocytic recycling, and retrograde transport of glycosyltransferases from the TGN to the Golgi; involved in organization of the ER, establishment of cell polarity, and morphogenesis; detected in highly purified mitochondria in high-throughput studies
YDR167W	*TAF10*	0.16	0.18	0.11	**0.15**	Chromatin organization	Subunit (145 kDa) of TFIID and SAGA complexes; involved in RNA polymerase II transcription initiation and in chromatin modification
YDR301W	*CFT1*	0.18	0.13	0.11	**0.14**	mRNA processing	RNA-binding subunit of the mRNA cleavage and polyadenylation factor; involved in poly(A) site recognition and required for both pre-mRNA cleavage and polyadenylation, 51% sequence similarity with mammalian AAUAA-binding subunit of CPSF
YDR339C	*FCF1*	0.15	0.18	0.19	**0.17**	rRNA processing	PINc domain endonuclease required for early cleavage of 35S pre-rRNA and maturation of 18S rRNA; component of the SSU (small subunit) processome involved in 40S ribosomal subunit biogenesis; copurifies with Faf1p
YDR429C	*TIF35*	0.18	0.12	0.11	**0.14**	Translation	eIF3g subunit of the eukaryotic translation initiation factor 3 (eIF3); subunit of the core complex of eIF3; is essential for translation; stimulates resumption of ribosomal scanning during translation reinitiation; eIF3 is also involved in programmed stop codon readthrough
YDR454C	*GUK1*	0.19	0.15	0.13	**0.16**	Cell wall	Guanylate kinase; converts GMP to GDP; required for growth and mannose outer chain elongation of cell wall N-linked glycoproteins
YDR489W	*SLD5*	0.17	0.21	0.14	**0.17**	DNA replication	Subunit of the GINS complex (Sld5p, Psf1p, Psf2p, Psf3p); complex is localized to DNA replication origins and implicated in assembly of the DNA replication machinery
YFR037C	*RSC8*	0.20	0.25	0.15	**0.20**	Chromatin organization	Component of the RSC chromatin remodeling complex; essential for viability and mitotic growth; homolog of SWI/SNF subunit Swi3p, but unlike Swi3p, does not activate transcription of reporters
YGL048C	*RPT6*	0.18	0.17	0.17	**0.17**	Protein degradation	ATPase of the 19S regulatory particle of the 26S proteasome; one of six ATPases of the regulatory particle; involved in the degradation of ubiquitinated substrates; bound by ubiquitin-protein ligases Ubr1p and Ufd4p; localized mainly to the nucleus throughout the cell cycle; protein abundance increases in response to DNA replication stress

YGL092W	*NUP145*	0.11	0.30	0.12	**0.18**	Nuclear transport	Essential protein with distinct roles in two nuclear pore subcomplexes; catalyzes its own proteolytic cleavage in vivo to generate a C-terminal fragment that is a structural component of the Nup84p subcomplex (with roles in NPC biogenesis and localization of genes to the nuclear periphery), and an N-terminal fragment that is one of several FG-nucleoporins within the NPC central core directly responsible for nucleocytoplasmic transport; homologous to human NUP98
YGL116W	*CDC20*	0.10	0.11	0.08	**0.10**	Cell cycle	Activator of anaphase-promoting complex/cyclosome (APC/C); APC/C is required for metaphase/anaphase transition; directs ubiquitination of mitotic cyclins, Pds1p, and other anaphase inhibitors; cell-cycle regulated; potential Cdc28p substrate; relative distribution to the nucleus increases upon DNA replication stress
YGL145W	*TIP20*	0.16	0.15	0.09	**0.14**	Golgi vesicle transport	Peripheral membrane protein required for COPI vesicle fusion to the ER; mediates Sey1p-independent homotypic ER fusion; prohibits back-fusion of COPII vesicles with the ER; forms a tethering complex with Sec39p and Dsl1p that interacts with ER SNAREs Sec20p and Use1p
YGL207W	*SPT16*	0.15	0.32	0.12	**0.20**	Chromatin organization	Subunit of the heterodimeric FACT complex (Spt16p-Pob3p); FACT associates with chromatin via interaction with Nhp6Ap and Nhp6Bp, and reorganizes nucleosomes to facilitate access to DNA by RNA and DNA polymerases; specifically required for diauxic shift-induced H2B deposition onto rDNA genes; mutations cause reduced nucleosome occupancy over highly transcribed regions; coregulates transcription with Mot1p through preinitiation complex assembly and nucleosome organization
YGL225W	*VRG4*	0.09	0.04	0.03	**0.06**	Protein glycosylation	Golgi GDP-mannose transporter; regulates Golgi function and glycosylation in Golgi; VRG4 has a paralog, HVG1, that arose from the whole genome duplication
YGR091W	*PRP31*	0.09	0.26	0.13	**0.16**	mRNA processing	Splicing factor; component of the U4/U6-U5 snRNP complex
YGR172C	*YIP1*	0.20	0.17	0.11	**0.16**	Golgi vesicle transport	Integral membrane protein; required for the biogenesis of ER-derived COPII transport vesicles; interacts with Yif1p and Yos1p; localizes to the Golgi, the ER, and COPII vesicles; human homolog YIPF5 can complement yeast yip1 mutant
YGR198w	*YPP1*	0.15	0.18	0.17	**0.17**	Protein targeting	Cargo-transport protein involved in endocytosis; interacts with phosphatidylinositol-4-kinase Stt4p; is required, along with Efr3p, for the assembly and recruitment of multiple copies of the kinase into phosphoinositide kinase (PIK) patches at the plasma membrane; positively regulates Stt4p; GFP-fusion protein localizes to the cytoplasm; YGR198W is an essential gene

YIL126W	*STH1*	0.08	0.15	0.11	**0.12**	Chromatin organization	ATPase component of the RSC chromatin remodeling complex; required for expression of early meiotic genes; promotes base excision repair in chromatin; essential helicase-related protein homologous to Snf2p
YJL097W	*PHS1*	0.11	0.11	0.08	**0.10**	Lipid metabolism	Essential 3-hydroxyacyl-CoA dehydratase of the ER membrane; involved in elongation of very long-chain fatty acids; evolutionarily conserved, similar to mammalian PTPLA and PTPLB; involved in sphingolipid biosynthesis and protein trafficking
YKL006C-A	*SFT1*	0.08	0.10	0.08	**0.09**	Golgi vesicle transport	Intra-Golgi v-SNARE; required for transport of proteins between an early and a later Golgi compartment
YKL165C	*MCD4*	0.16	0.23	0.15	**0.18**	GPI biosynthesis	Protein involved in GPI anchor synthesis; multimembrane-spanning protein that localizes to the endoplasmic reticulum; highly conserved among eukaryotes; GPI stands for glycosylphosphatidylinositol
YKL172W	*EBP2*	0.15	0.18	0.19	**0.17**	rRNA processing	Required for 25S rRNA maturation and 60S ribosomal subunit assembly; localizes to the nucleolus and in foci along nuclear periphery; constituent of 66S pre-ribosomal particles; cooperates with Rrs1p and Mps3p to mediate telomere clustering by binding Sir4p, but is not involved in telomere tethering
YKR068C	*BET3*	0.08	0.13	0.18	**0.13**	Golgi vesicle transport	Core component of transport protein particle (TRAPP) complexes I-III; TRAPP complexes are related multimeric guanine nucleotide-exchange factors for the GTPase Ypt1, regulating ER-Golgi traffic (TRAPPI), intra-Golgi traffic (TRAPPII), endosome-Golgi traffic (TRAPPII and III) and autophagy (TRAPPIII); hydrophilic homodimeric protein that acts in conjunction with SNARE proteins in targeting and fusion of ER to Golgi transport vesicles
YLR033W	*RSC58*	0.10	0.30	0.20	**0.20**	Chromatin organization	Component of the RSC chromatin remodeling complex; RSC functions in transcriptional regulation and elongation, chromosome stability, and establishing sister chromatid cohesion; involved in telomere maintenance
YLR276C	*DBP9*	0.16	0.26	0.18	**0.20**	rRNA processing	DEAD-box protein required for 27S rRNA processing; exhibits DNA, RNA and DNA/RNA helicase activities; ATPase activity shows preference for DNA over RNA; DNA helicase activity abolished by mutation in RNA-binding domain
YLR459W	*GAB1*	0.19	0.09	0.08	**0.12**	GPI biosynthesis	GPI transamidase subunit; involved in attachment of glycosylphosphatidylinositol (GPI) anchors to proteins; may have a role in recognition of the attachment signal or of the lipid portion of GPI

YMR079W	*SEC14*	0.16	0.21	0.07	**0.14**	Golgi vesicle transport	Phosphatidylinositol/phosphatidylcholine transfer protein; involved in regulating PtdIns, PtdCho, and ceramide metabolism, products of which regulate intracellular transport and UPR; has a role in localization of lipid raft proteins; functionally homologous to mammalian PITPs; SEC14 has a paralog, YKL091C, that arose from the whole genome duplication
YMR149W	*SWP1*	0.06	0.31	0.16	**0.18**	Protein glycosylation	Delta subunit of the oligosaccharyl transferase glycoprotein complex; complex is required for N-linked glycosylation of proteins in the endoplasmic reticulum
YMR200W	*ROT1*	0.07	0.14	0.13	**0.11**	Protein glycosylation	Molecular chaperone involved in protein folding in ER; mutation causes defects in cell wall synthesis and lysis of autophagic bodies, suppresses tor2 mutations, and is synthetically lethal with kar2-1 and with rot2 mutations; involved in N-linked glycosylation and O-mannosylation; transmembrane helix Ser250 is essential for Rot1p to interact with other membrane components and exert its functional role, avoiding exposure of Ser H-bonding group at lipid-exposed surface
YMR314W	*PRE5*	0.15	0.22	0.16	**0.18**	Protein degradation	Alpha 6 subunit of the 20S proteasome; protein abundance increases in response to DNA replication stress
YNL061W	*NOP2*	0.12	0.19	0.13	**0.15**	rRNA processing	rRNA m5C methyltransferase; methylates cytosine at position 2870 of 25S rRNA; has an essential function independent of rRNA methylation; contains seven beta-strand methyltransferase motif; essential for processing and maturation of 27S pre-rRNA and large ribosomal subunit biogenesis; localized to the nucleolus; constituent of 66S pre-ribosomal particles; rRNA methylation defect and lethality are functionally complemented by human NOP2, a gene upregulated in cancer
YNL110c	*NOP15*	0.18	0.18	0.22	**0.19**	rRNA processing	Constituent of 66S pre-ribosomal particles; involved in 60S ribosomal subunit biogenesis; localizes to both nucleolus and cytoplasm
YNL118C	*DCP2*	0.16	0.16	0.10	**0.14**	mRNA processing	Catalytic subunit of Dcp1p-Dcp2p decapping enzyme complex; removes 5' cap structure from mRNAs prior to their degradation; also enters nucleus and positively regulates transcription initiation; nudix hydrolase family member; forms cytoplasmic foci upon DNA replication stress; human homolog DCP2 complements yeast dcp2 thermosensitive mutant
YNL272C	*SEC2*	0.16	0.07	0.09	**0.11**	Golgi vesicle transport	Guanyl-nucleotide exchange factor for the small G-protein Sec4p; essential for post-Golgi vesicle transport and for autophagy; associates with the exocyst, via exocyst subunit Sec15p, on secretory vesicles
YOL038W	*PRE6*	0.17	0.21	0.17	**0.18**	Protein degradation	Alpha 4 subunit of the 20S proteasome; may replace alpha 3 subunit (Pre9p) under stress conditions to create a more active proteasomal isoform; GFP-fusion protein relocates from cytosol to the mitochondrial surface upon oxidative stress

YOL077c	*BRX1*	0.18	0.14	0.13	**0.15**	rRNA processing	Nucleolar protein; constituent of 66S pre-ribosomal particles; depletion leads to defects in rRNA processing and a block in the assembly of large ribosomal subunits; possesses a sigma(70)-like RNA-binding motif
YOL120C	*RPL18A*	0.20	0.19	0.11	**0.17**	Translation	Ribosomal 60S subunit protein L18A; intron of RPL18A pre-mRNA forms stem-loop structures that are a target for Rnt1p cleavage leading to degradation; homologous to mammalian ribosomal protein L18, no bacterial homolog; RPL18A has a paralog, RPL18B, that arose from the whole genome duplication
YOR103C	*OST2*	0.05	0.06	0.08	**0.07**	Protein glycosylation	Epsilon subunit of the oligosaccharyltransferase complex; located in the ER lumen; catalyzes asparagine-linked glycosylation of newly synthesized proteins
YOR122C	*PFY1*	0.09	0.06	0.04	**0.06**	Cytoskeleton organization	Profilin; binds actin, phosphatidylinositol 4,5-bisphosphate, and polyproline regions; involved in cytoskeleton organization; required for normal timing of actin polymerization in response to thermal stress; protein abundance increases in response to DNA replication stress; highly conserved protein; human PFN1 (profilin 1) complements temperature sensitive pfy1 mutants, PFN1 mutations are a rare cause of ALS
YPL082C	*MOT1*	0.11	0.38	0.07	**0.18**	Transcription from RNA pol II	Essential protein involved in regulation of transcription; removes Spt15p (TBP) from DNA via its C-terminal ATPase activity; may have a role in ensuring that soluble TBP is available to bind TATA-less promoters; forms a complex with TBP that binds TATA DNA with high affinity but with altered specificity; the Mot1p-Spt15p-DNA ternary complex contains unbent DNA; coregulates transcription with Spt16p through assembly of preinitiation complex and organization of nucleosomes
YPL210C	*SRP72*	0.14	0.14	0.11	**0.13**	Protein targeting	Core component of the signal recognition particle (SRP); the SRP is a ribonucleoprotein (RNP) complex that functions in targeting nascent secretory proteins to the endoplasmic reticulum (ER) membrane
YPL243W	*SRP68*	0.15	0.32	0.09	**0.19**	Protein targeting	Core component of the signal recognition particle (SRP) complex; SRP complex functions in targeting nascent secretory proteins to the endoplasmic reticulum (ER) membrane; relocalizes from cytoplasm to the nuclear periphery upon DNA replication stress
YPR034W	*ARP7*	0.20	0.20	0.17	**0.19**	Chromatin organization	Component of both the SWI/SNF and RSC chromatin remodeling complexes; actin-related protein involved in transcriptional regulation
