# Supplementary material for: Systematic Identification of Essential Genes Required for Yeast Cell Wall Integrity: Involvement of the RSC Remodelling Complex
Source: J Fungi (Basel). 2022 Jul 8;8(7):718. doi: 10.3390/jof8070718 (PMC9323250; doi:10.3390/jof8070718)
Supplement: Supplementary file 1 [file jof-08-00718-s001.zip › Supplemental Table S4.pdf]

Table S4. Positive hits from the Mlp1-GFP expression screening. For each mutant strain, data from three independent experiments (exp) and their corresponding mean value are shown. Mutants selected by "low percentage" of GFP positive cells under stress conditions are highlighted in grey. See Table S3 for additional information.

**Mutated ORF**	**Mutated gene**	**Mlp1-GFP ratio CW+/CW- (exp 1)**	**Mlp1-GFP ratio CW+/CW- (exp 2)**	**Mlp1-GFP ratio CW+/CW- (exp 3)**	**Mean**	**Biological process**	**Description from the *Saccharomyces Genome Database***
YAL038W	*CDC19*	14.84	7.95	4.63	**9.14**	Carbohydrate metabolism	Pyruvate kinase; functions as a homotetramer in glycolysis to convert phosphoenolpyruvate to pyruvate, the input for aerobic (TCA cycle) or anaerobic (glucose fermentation) respiration; regulated via allosteric activation by fructose bisphosphate; CDC19 has a paralog, PYK2, that arose from the whole genome duplication
YAR008W	*SEN34*	9.19	2.43	1.00	**4.21**	tRNA processing	Subunit of the tRNA splicing endonuclease; tRNA splicing endonuclease (Sen complex) is composed of Sen2p, Sen15p, Sen34p, and Sen54p; Sen complex also cleaves the CBP1 mRNA at the mitochondrial surface; Sen34p contains the active site for tRNA 3' splice site cleavage and has similarity to Sen2p and to Archaeal tRNA splicing endonuclease
YBL018C	*POP8*	2.58	0.58	1.02	**1.39**	rRNA processing	Subunit of both RNase MRP and nuclear RNase P; RNase MRP cleaves pre-rRNA, while nuclear RNase P cleaves tRNA precursors to generate mature 5' ends and facilitates turnover of nuclear RNAs; relocalizes to the cytosol in response to hypoxia
YBL076C	*ILS1*	2.41	8.81	8.13	**6.45**	Amino acid metabolism	Cytoplasmic isoleucine-tRNA synthetase; target of the G1-specific inhibitor reveromycin A
YBR079C	*RPG1*	5.44	1.23	0.60	**2.42**	Translation	eIF3a subunit of the eukaryotic translation initiation factor 3 (eIF3); subunit of the core complex of eIF3; essential for translation; part of a Prt1p-Rpg1p-Nip1p subcomplex that stimulates binding of mRNA and tRNA(i)Met to ribosomes; involved in translation reinitiation; eIF3 is also involved in programmed stop codon readthrough
YBR155W	*CNS1*	10.68	1.41	0.60	**4.23**	Protein folding	TPR-containing co-chaperone; binds both Hsp82p (Hsp90) and Ssa1p (Hsp70); stimulates ATPase activity of Ssa1p; ts mutants reduce Hsp82p function, overexpression suppresses phenotypes of HSP82 ts allele and cpr7 deletion; human homolog TTC4 complements yeast cns1 mutant
YCR035C	*RRP43*	10.67	4.86	2.12	**5.88**	Exosome component	Exosome non-catalytic core component; involved in 3'-5' RNA processing and degradation in both the nucleus and the cytoplasm; has similarity to E. coli RNase PH and to human hRrp43p (OIP2, EXOSC8); protein abundance increases in response to DNA replication stress

YDL007W	*RPT2*	0.14	0.22	0.29	**0.22**	Protein degradation	ATPase of the 19S regulatory particle of the 26S proteasome; one of six ATPases of the regulatory particle; involved in the degradation of ubiquitinated substrates; required for normal peptide hydrolysis by the core 20S particle; N-myristoylation of Rpt2p at Gly2 is involved in regulating the proper intracellular distribution of proteasome activity by controlling the nuclear localization of the 26S proteasome
YDL015C	*TSC13*	3.56	1.83	1.17	**2.19**	Lipid metabolism	Enoyl reductase; catalyzes last step in each cycle of very long chain fatty acid elongation; localizes to ER, highly enriched in a structure marking nuclear-vacuolar junctions; coimmunoprecipitates with elongases Elo2p and Elo3p; protein increases in abundance and relative distribution to ER foci increases upon DNA replication stress; human homolog TECR implicated in nonsyndromic mental retardation, can complement yeast mutant
YDL055C	*PSA1*	0.23	0.42	0.11	**0.25**	Protein glycosilation	GDP-mannose pyrophosphorylase (mannose-1-phosphate guanyltransferase); synthesizes GDP-mannose from GTP and mannose-1-phosphate in cell wall biosynthesis; required for normal cell wall structure
YDL092W	*SRP14*	0.14	0.18	0.19	**0.17**	Protein targeting	Signal recognition particle (SRP) subunit; interacts with the RNA component of SRP to form the Alu domain, which is the region of SRP responsible for arrest of nascent chain elongation during membrane targeting; homolog of mammalian SRP14
YDL143W	*CCT4*	0.15	0.13	0.13	**0.14**	Cytoskeleton organization	Subunit of the cytosolic chaperonin Cct ring complex; related to Tcp1p, required for the assembly of actin and tubulins in vivo
YDR023W	*SES1*	2.54	0.41	0.52	**1.16**	Amino acid metabolism	Cytosolic seryl-tRNA synthetase; class II aminoacyl-tRNA synthetase that aminoacylates tRNA(Ser), displays tRNA-dependent amino acid recognition which enhances discrimination of the serine substrate, interacts with peroxin Pex21p
YDR044W	*HEM13*	0.93	1.22	1.11	**1.09**	Cofactor metabolism	Coproporphyrinogen III oxidase; oxygen-requiring enzyme that catalyzes sixth step in heme biosynthetic pathway; transcription is repressed by oxygen and heme (via Rox1p and Hap1p); human homolog CPOX can complement yeast mutant and allow growth of haploid null after sporulation of a heterozygous diploid
YDR050C	*TPI1*	0.22	0.26	0.23	**0.24**	Carbohydrate metabolism	Triose phosphate isomerase, abundant glycolytic enzyme; mRNA half-life is regulated by iron availability; transcription is controlled by activators Reb1p, Gcr1p, and Rap1p through binding sites in the 5' non-coding region; inhibition of Tpi1p activity by PEP (phosphoenolpyruvate) stimulates redox metabolism in respiring cells; E104D mutation in human homolog TPI1 causes a rare autosomal disease; human TPI1 can complement yeast null mutant
YDR054C	*CDC34*	0.24	0.16	0.27	**0.22**	Protein degradation	Ubiquitin-conjugating enzyme (E2); catalytic subunit of SCF ubiquitin-protein ligase complex (together with Skp1p, Rbx1p, Cdc53p, and an F-box protein) that regulates cell cycle progression by targeting key substrates for degradation; protein abundance increases in response to DNA replication stress; human CDC34 functionally complements the thermosensitivity of the cdc34-2 mutant

YDR060W	*MAK21*	0.98	1.09	1.98	**1.35**	Ribosome biogenesis	Constituent of 66S pre-ribosomal particles; required for large (60S) ribosomal subunit biogenesis; acts as part of a Mak21p-Noc2p-Rrp5p module that associates with nascent pre-rRNA during transcription and has a role in bigenesis of the large ribosomal subunit; involved in nuclear export of pre-ribosomes; required for maintenance of dsRNA virus; homolog of human CAATT-binding protein
YDR062W	*LCB2*	0.24	0.18	0.34	**0.25**	Lipid metabolism	Component of serine palmitoyltransferase; responsible along with Lcb1p for the first committed step in sphingolipid synthesis, which is the condensation of serine with palmitoyl-CoA to form 3-ketosphinganine
YDR168w	*CDC37*	0.80	1.69	0.40	**0.96**	Protein folding	Essential Hsp90p co-chaperone; necessary for passage through the START phase of the cell cycle; stabilizes protein kinase nascent chains and participates along with Hsp90p in their folding
YDR196C	*CAB5*	3.90	1.77	2.22	**2.63**	Cofactor metabolism	Subunit of the CoA-Synthesizing Protein Complex (CoA-SPC); subunits of this complex are: Cab2p, Cab3p, Cab4p, Cab5p, Sis2p and Vhs3p; probable dephospho-CoA kinase (DPCK) that catalyzes the last step in coenzyme A biosynthesis; null mutant lethality is complemented by human homolog DCAKD and by E. coli coaE (encoding DPCK); detected in purified mitochondria in high-throughput studies; also localized to lipid droplets
YDR228C	*PCF11*	5.72	0.95	1.43	**2.70**	mRNA processing	mRNA 3' end processing factor; essential component of cleavage and polyadenylation factor IA (CF IA), involved in pre-mRNA 3' end processing and in transcription termination; binds C-terminal domain of largest subunit of RNA pol II (Rpo21p); required for gene looping; relocalizes to the cytosol in response to hypoxia
YDR280W	*RRP45*	14.03	21.27	0.41	**11.90**	Exosome component	Exosome non-catalytic core component; involved in 3'-5' RNA processing and degradation in both the nucleus and the cytoplasm; has similarity to E. coli RNase PH and to human hRrp45p (PM/SCL-75, EXOSC9); protein abundance increases in response to DNA replication stress
YDR412W	*RRP17*	11.06	10.02	10.01	**10.36**	rRNA processing	Component of the pre-60S pre-ribosomal particle; required for cell viability under standard (aerobic) conditions but not under anaerobic conditions; exonuclease required for 5' end processing of pre-60S ribosomal RNA
YDR464W	*SPP41*	7.87	5.65	12.28	**8.60**	Unknown	Protein of unknown function; involved in negative regulation of expression of spliceosome components PRP4 and PRP3; relocalizes to the cytosol in response to hypoxia
YDR531W	*CAB1*	0.92	0.52	0.34	**0.59**	Cofactor metabolism	Pantothenate kinase, ATP:D-pantothenate 4'-phosphotransferase; catalyzes the first committed step in the universal biosynthetic pathway for synthesis of coenzyme A (CoA); transcriptionally regulated by Upc2p via a sterol response element
YEL055C	*POL5*	4.68	0.95	0.66	**2.10**	rRNA synthesis	DNA Polymerase phi; has sequence similarity to the human MybBP1A and weak sequence similarity to B-type DNA polymerases, not required for chromosomal DNA replication; required for the synthesis of rRNA

YER013W	*PRP22*	6.91	1.49	2.09	**3.50**	mRNA processing	DEAH-box RNA-dependent ATPase/ATP-dependent RNA helicase; associates with lariat intermediates before the second catalytic step of splicing; mediates ATP-dependent mRNA release from the spliceosome and unwinds RNA duplexes; required for proofreading the exon ligation reaction
YFL035C-A	*MOB2*	0.11	0.36	0.17	**0.21**	Signaling	Activator of Cbk1p kinase; component of the RAM signaling network that regulates cellular polarity and morphogenesis; activation of Cbk1p facilitates the Ace2p-dependent daughter cell-specific transcription of genes involved in cell separation; similar to Mob1p
YFR004W	*RPN11*	8.06	1.88	2.25	**4.06**	Protein degradation	Metalloprotease subunit of 19S regulatory particle; part of 26S proteasome lid; couples the deubiquitination and degradation of proteasome substrates; involved, independent of catalytic activity, in fission of mitochondria and peroxisomes; protein abundance increases in response to DNA replication stress
YFR031C	*SMC2*	1.79	1.04	1.15	**1.33**	Cell cycle	Subunit of the condensin complex; condensin reorganizes chromosomes during both mitosis and meiosis; essential SMC chromosomal ATPase family member that forms a subcomplex with Smc2p that has ATP-hydrolyzing and DNA-binding activity, but other condensin subunits are required for chromatin binding; required for clustering of tRNA genes at the nucleolus
YGL001C	*ERG26*	0.20	0.17	0.21	**0.19**	Lipid metabolism	C-3 sterol dehydrogenase; catalyzes the second of three steps required to remove two C-4 methyl groups from an intermediate in ergosterol biosynthesis; human homolog NSDHL implicated in CK syndrome, and can complement yeast null mutant; molecular target of natural product and antifungal compound FR171456
YGL061C	*DUO1*	2.96	0.59	0.14	**1.23**	Cytoskeleton organization	Essential subunit of the Dam1 complex (aka DASH complex); cooperates with Dam1p to connect the DASH complex with microtubules (MT); couples kinetochores to the force produced by MT depolymerization thereby aiding in chromosome segregation; is transferred to the kinetochore prior to mitosis
YGL068W	*MNP1*	8.50	5.65	3.53	**5.89**	Translation	Mitochondrial ribosomal protein of the large subunit; has similarity to E. coli L7/L12 and human MRPL7 ribosomal proteins; associates with the mitochondrial nucleoid; required for normal respiratory growth
YGL092W	*NUP145*	1.36	1.02	5.42	**2.60**	Nuclear transport	Essential protein with distinct roles in two nuclear pore subcomplexes; catalyzes its own proteolytic cleavage in vivo to generate a C-terminal fragment that is a structural component of the Nup84p subcomplex (with roles in NPC biogenesis and localization of genes to the nuclear periphery), and an N-terminal fragment that is one of several FG-nucleoporins within the NPC central core directly responsible for nucleocytoplasmic transport; homologous to human NUP98
YGL103W	*RPL28*	0.16	0.24	0.23	**0.21**	Translation	Ribosomal 60S subunit protein L28; homologous to mammalian ribosomal protein L27A and bacterial L15; may have peptidyl transferase activity; can mutate to cycloheximide resistance

YGL137W	*SEC27*	0.16	0.29	0.26	**0.24**	Golgi vesicle transport	Essential beta'-coat protein of the COPI coatomer; involved in ER-to-Golgi and Golgi-to-ER transport; contains WD40 domains that mediate cargo selective interactions; 45% sequence identity to mammalian beta'-COP
YGR002C	*SWC4*	0.11	0.23	0.28	**0.20**	Chromatin organization	Component of the Swr1p complex that incorporates Htz1p into chromatin; component of the NuA4 histone acetyltransferase complex
YGR091W	*PRP31*	6.75	2.55	3.93	**4.41**	mRNA processing	Splicing factor; component of the U4/U6-U5 snRNP complex
YGR116W	*SPT6*	3.48	0.65	0.91	**1.68**	Chromatin organization	Nucleosome remodeling protein; functions in various aspects of transcription, chromatin maintenance, and RNA processing; required for the maintenance of chromatin structure during transcription in order to inhibit transcription from promoters within the coding region; required for H3K36 trimethylation but not dimethylation by Set2p
YGR128C	*UTP8*	12.42	9.15	5.56	**9.04**	Nuclear transport	Nucleolar protein required for export of tRNAs from the nucleus; also copurifies with the small subunit (SSU) processome containing the U3 snoRNA that is involved in processing of pre-18S rRNA
YGR158C	*MTR3*	10.94	3.68	4.55	**6.39**	Exosome component	Exosome non-catalytic core component; involved in 3'-5' RNA processing and degradation in both the nucleus and the cytoplasm; has similarity to E. coli RNase PH and to human hMtr3p (EXOSC6)
YGR175C	*ERG1*	4.76	12.19	22.90	**13.28**	Lipid metabolism	Squalene epoxidase; catalyzes the epoxidation of squalene to 2,3-oxidosqualene; plays an essential role in the ergosterol-biosynthesis pathway and is the specific target of the antifungal drug terbinafine; human SQLE functionally complements the lethality of the erg1 null mutation
YGR274C	*TAF1*	3.35	1.13	3.12	**2.53**	Transcription from RNA pol II	TFIID subunit, involved in RNA pol II transcription initiation; possesses in vitro histone acetyltransferase activity but its role in vivo appears to be minor; involved in promoter binding and G1/S progression; relocalizes to the cytosol in response to hypoxia
YHR019C	*DED81*	9.86	7.53	12.73	**10.04**	Amino acid metabolism	Cytosolic asparaginyl-tRNA synthetase; required for protein synthesis, catalyzes the specific attachment of asparagine to its cognate tRNA
YHR058C	*MED6*	14.54	14.49	14.26	**14.43**	Transcription from RNA pol II	Subunit of the RNA polymerase II mediator complex; associates with core polymerase subunits to form the RNA polymerase II holoenzyme; essential for transcriptional regulation; protein abundance increases in response to DNA replication stress
YHR089C	*GAR1*	14.06	4.47	4.93	**7.82**	rRNA processing	Protein component of the H/ACA snoRNP pseudouridylase complex; involved in the modification and cleavage of the 18S pre-rRNA
YHR196w	*UTP9*	1.05	0.80	0.00	**0.62**	rRNA processing	Nucleolar protein; component of the small subunit (SSU) processome containing the U3 snoRNA that is involved in processing of pre-18S rRNA
YIL021W	*RPB3*	14.00	0.74	1.28	**5.34**	Transcription from RNA pol II	RNA polymerase II third largest subunit B44; part of central core; similar to prokaryotic alpha subunit
YIL061C	*SNP1*	3.29	2.22	9.34	**4.95**	mRNA processing	Component of U1 snRNP required for mRNA splicing via spliceosome; substrate of the arginine methyltransferase Hmt1p; may interact with poly(A) polymerase to regulate polyadenylation; homolog of human U1 70K protein; protein abundance increases in response to DNA replication stress

YIL078w	*THS1*	2.72	0.12	0.77	**1.20**	Amino acid metabolism	Threonyl-tRNA synthetase; essential cytoplasmic protein; human homolog TARS can complement yeast null mutant
YJL194W	*CDC6*	0.13	0.26	0.10	**0.16**	DNA replication	Essential ATP-binding protein required for DNA replication; component of the pre-replicative complex (pre-RC) which requires ORC to associate with chromatin and is in turn required for Mcm2-7p DNA association; homologous to S. pombe Cdc18p; relocalizes from nucleus to cytoplasm upon DNA replication stress; degraded in response to plasma membrane stress
YJR072C	*NPA3*	1.38	0.99	1.12	**1.16**	Protein targeting	Member of the conserved GPN-loop GTPase family; has a role in transport of RNA polymerase II to the nucleus; exhibits GTP-dependent binding to PolII; has ATPase activity; involved in sister chromatid cohesion; phosphorylated by the Pcl1p-Pho85p kinase complex; human homolog XAB1 interacts with human RNA polymerase II; protein abundance increases in response to DNA replication stress
YJR123W	*RPS5*	10.52	12.39	12.46	**11.79**	Translation	Protein component of the small (40S) ribosomal subunit; least basic of non-acidic ribosomal proteins; phosphorylated in vivo; essential for viability; homologous to mammalian ribosomal protein S5 and bacterial S7
YKL006C-A	*SFT1*	1.10	6.62	2.69	**3.47**	Golgi vesicle transport	Intra-Golgi v-SNARE; required for transport of proteins between an early and a later Golgi compartment
YKL210w	*UBA1*	8.10	7.93	2.96	**6.33**	Protein degradation	Ubiquitin activating enzyme (E1); involved in ubiquitin-mediated protein degradation and essential for viability; protein abundance increases in response to DNA replication stress
YLL035W	*GRC3*	0.23	0.21	0.30	**0.24**	rRNA processing	Polynucleotide kinase present on rDNA; required for efficient transcription termination by RNA polymerase I; functions with Las1p in a conserved mechanism to modulate rRNA processing and ribosome biogenesis; required for cell growth; mRNA is cell-cycle regulated
YLR033W	*RSC58*	0.22	0.24	0.27	**0.24**	Chromatin organization	Component of the RSC chromatin remodeling complex; RSC functions in transcriptional regulation and elongation, chromosome stability, and establishing sister chromatid cohesion; involved in telomere maintenance
YLR060W	*FRS1*	15.01	14.65	11.61	**13.76**	Amino acid metabolism	Beta subunit of cytoplasmic phenylalanyl-tRNA synthetase; forms a tetramer with Frs2p to generate active enzyme; able to hydrolyze mis-aminoacylated tRNA-Phe, which could contribute to translational quality control
YLR071C	*RGR1*	2.47	0.60	0.54	**1.20**	Transcription from RNA pol II	Subunit of the RNA polymerase II mediator complex; associates with core polymerase subunits to form the RNA polymerase II holoenzyme; required for glucose repression, HO repression, RME1 repression and sporulation
YLR088W	*GAA1*	7.44	0.75	2.35	**3.51**	GPI biosyntesis	Subunit of the GPI:protein transamidase complex; removes the GPI-anchoring signal and attaches GPI (glycosylphosphatidylinositol) to proteins in the ER; human homolog GPAA1 can complement growth defects of yeast thermosensitive mutant at restrictive temperature
YLR140W	*YLR140W*	16.40	8.60	7.83	**10.94**	Unknown	Dubious open reading frame unlikely to encode a functional protein; overlaps essential RRN5 gene which encodes a member of the UAF transcription factor involved in transcription of rDNA by RNA polymerase I

YLR274W	*MCM5*	0.22	0.16	0.11	**0.16**	DNA replication	Component of the Mcm2-7 hexameric helicase complex; MCM complex is important for priming origins of DNA replication in G1 and becomes an active ATP-dependent helicase that promotes DNA melting and elongation when activated by Cdc7p-Dbf4p in S-phase
YLR355C	*ILV5*	17.08	8.69	11.16	**12.31**	Amino acid metabolism	Acetohydroxyacid reductoisomerase and mtDNA binding protein; involved in branched-chain amino acid biosynthesis and maintenance of wild-type mitochondrial DNA; found in mitochondrial nucleoids
YML015C	*TAF11*	5.62	1.29	2.17	**3.03**	Transcription from RNA pol II	TFIID subunit (40 kDa); involved in RNA polymerase II transcription initiation, similar to histone H3 with atypical histone fold motif of Spt3-like transcription factors
YML023C	*NSE5*	12.48	1.62	1.52	**5.21**	DNA replication	Component of the SMC5-SMC6 complex; this complex plays a key role in the removal of X-shaped DNA structures that arise between sister chromatids during DNA replication and repair
YML065W	*ORC1*	3.25	4.29	1.88	**3.14**	DNA replication	Largest subunit of the origin recognition complex; involved in directing DNA replication by binding to replication origins; also involved in transcriptional silencing; exhibits ATPase activity; ORC1 has a paralog, SIR3, that arose from the whole genome duplication
YML085C	*TUB1*	7.49	10.95	9.49	**9.31**	Cytoskeleton organization	Alpha-tubulin; associates with beta-tubulin (Tub2p) to form tubulin dimer, which polymerizes to form microtubules; relative distribution to nuclear foci increases upon DNA replication stress; TUB1 has a paralog, TUB3, that arose from the whole genome duplication
YML127W	*RSC9*	0.22	0.28	0.25	**0.25**	Chromatin organization	Component of the RSC chromatin remodeling complex; DNA-binding protein involved in the synthesis of rRNA and in transcriptional repression and activation of genes regulated by the Target of Rapamycin (TOR) pathway
YMR005W	*TAF4*	4.50	9.26	13.33	**9.03**	Transcription from RNA pol II	TFIID subunit (48 kDa); involved in RNA polymerase II transcription initiation; potential Cdc28p substrate
YMR146C	*TIF34*	13.13	3.26	6.05	**7.48**	Translation	eIF3i subunit of the eukaryotic translation initiation factor 3 (eIF3); subunit of the core complex of eIF3; essential for translation; stimulates rate of ribosomal scanning during translation reinitiation; eIF3 is also involved in programmed stop codon readthrough
YMR203W	*TOM40*	4.17	7.55	7.83	**6.52**	Protein targeting	Component of the TOM (translocase of outer membrane) complex; responsible for recognition and initial import steps for all mitochondrially directed proteins; constitutes the core element of the protein conducting pore; pre-Tom40p is phosphorylated by PKA, which impairs its import into mitochondria under non-respiratory conditions
YMR236W	*TAF9*	19.86	2.94	9.47	**10.76**	Chromatin organization	Subunit (17 kDa) of TFIID and SAGA complexes; involved in RNA polymerase II transcription initiation and in chromatin modification, similar to histone H3
YMR290C	*HAS1*	10.40	11.75	10.59	**10.91**	Ribosome biogenesis	ATP-dependent RNA helicase; involved in the biogenesis of 40S and 60S ribosome subunits; localizes to both the nuclear periphery and nucleolus; highly enriched in nuclear pore complex fractions; constituent of 66S pre-ribosomal particles

YMR314W	*PRE5*	0.24	0.31	0.22	**0.25**	Protein degradation	Alpha 6 subunit of the 20S proteasome; protein abundance increases in response to DNA replication stress
YNL113W	*RPC19*	8.92	3.57	13.02	**8.50**	Transcription from RNA pol III	RNA polymerase subunit AC19; common to RNA polymerases I and III
YNL131W	*TOM22*	0.43	6.44	1.12	**2.66**	Protein targeting	Component of the TOM (Translocase of Outer Membrane) complex; responsible for initial import of mitochondrially directed proteins; mediates interaction between TOM and TIM complexes and acts as a receptor for precursor proteins
YNL149C	*PGA2*	2.83	1.76	26.56	**10.38**	Cell wall	Essential protein required for maturation of Gas1p and Pho8p; involved in protein trafficking; GFP-fusion protein localizes to the ER and YFP-fusion protein to the nuclear envelope-ER network; null mutants have a cell separation defect
YNL151C	*RPC31*	13.96	9.96	11.08	**11.67**	Transcription from RNA pol III	RNA polymerase III subunit C31
YNL207W	*RIO2*	6.37	1.59	1.04	**3.00**	rRNA processing	Essential serine kinase involved in the processing of 20S pre-rRNA; involved in the processing of the 20S pre-rRNA into mature 18S rRNA; has similarity to Rio1p
YNL247W	*YNL247W*	2.11	2.62	3.07	**2.60**	Amino acid metabolism	Cysteinyl-tRNA synthetase; may interact with ribosomes, based on co-purification experiments; human gene CARS allows growth of the yeast haploid null mutant after sporulation of a heterozygous diploid
YOL038W	*PRE6*	0.22	0.25	0.29	**0.25**	Protein degradation	Alpha 4 subunit of the 20S proteasome; may replace alpha 3 subunit (Pre9p) under stress conditions to create a more active proteasomal isoform; GFP-fusion protein relocates from cytosol to the mitochondrial surface upon oxidative stress
YOL094C	*RFC4*	1.59	4.16	9.70	**5.15**	DNA replication	Subunit of heteropentameric Replication factor C (RF-C); which is a DNA binding protein and ATPase that acts as a clamp loader of the proliferating cell nuclear antigen (PCNA) processivity factor for DNA polymerases delta and epsilon; relocalizes to the cytosol in response to hypoxia
YOL133W	*HRT1*	12.94	3.69	10.62	**9.08**	Protein degradation	RING-H2 domain core subunit of multiple ubiquitin ligase complexes; subunit of Skp1-Cullin-F-box (SCF) that tethers the Cdc34p (E2) and Cdc53p (cullin) SCF subunits, and is required for degradation of Gic2p, Far1p, Sic1p and Cln2p; subunit of the Rtt101p-Mms1p-Mms22p ubiquitin ligase that stabilizes replication forks after DNA lesions; subunit of the Cul3p-Elc1p-Ela1p ubiquitin ligase involved in Rpb1p degradation as part of transcription-coupled repair
YOL142W	*RRP40*	12.77	20.02	12.08	**14.96**	Exosome component	Exosome non-catalytic core component; involved in 3'-5' RNA processing and degradation in both the nucleus and the cytoplasm; predicted to contain both S1 and KH RNA binding domains; mutations in the human homolog, EXOSC3, cause pontocerebellar hypoplasia with motor neuron degeneration
YOR116C	*RPO31*	11.89	0.27	8.06	**6.74**	Transcription from RNA pol III	RNA polymerase III largest subunit C160; part of core enzyme; similar to bacterial beta-prime subunit and to RPA190 and RPO21

YOR204W	*DED1*	12.07	2.49	4.65	**6.40**	mRNA processing	ATP-dependent DEAD (Asp-Glu-Ala-Asp)-box RNA helicase; required for translation initiation of all yeast mRNAs; binds to mRNA cap-associated factors, and binding stimulates Ded1p RNA-dependent ATPase activity; mutation in human homolog DBY is associated with male infertility; human homolog DDX3X complements ded1 null mutation; DED1 has a paralog, DBP1, that arose from the whole genome duplication
YOR210W	*RPB10*	5.27	1.85	2.05	**3.06**	Transcription from RNA pol II	RNA polymerase subunit ABC10-beta; common to RNA polymerases I, II, and III
YOR236W	*DFR1*	1.66	1.75	1.63	**1.68**	Cofactor metabolism	Dihydrofolate reductase involved in tetrahydrofolate biosynthesis; required for respiratory metabolism; mutation is functionally complemented by human DHFR
YOR272W	*YTM1*	0.78	2.08	0.56	**1.14**	Ribosome biogenesis	Constituent of 66S pre-ribosomal particles; forms a complex with Nop7p and Erb1p that is required for maturation of the large ribosomal subunit; has seven C-terminal WD repeats
YOR281C	*PLP2*	0.18	0.44	0.14	**0.25**	Protein folding	Protein that interacts with the CCT complex to stimulate actin folding; has similarity to phosducins; null mutant lethality is complemented by mouse phosducin-like protein MgcPhLP; CCT is short for chaperonin containing TCP-1; essential gene
YOR361C	*PRT1*	1.33	0.76	0.88	**0.99**	Translation	eIF3b subunit of the eukaryotic translation initiation factor 3 (eIF3); subunit of the core complex of eIF3; essential for translation; part of a subcomplex (Prt1p-Rpg1p-Nip1p) that stimulates binding of mRNA and tRNA(i)Met to ribosomes; eIF3 is also involved in programmed stop codon readthrough
YPL010W	*RET3*	4.78	3.30	0.49	**2.86**	Golgi vesicle transport	Zeta subunit of the coatomer complex (COPI); COPI coats Golgi-derived transport vesicles; involved in retrograde transport between Golgi and ER
YPL011C	*TAF3*	3.08	2.15	2.25	**2.49**	Transcription from RNA pol II	TFIID subunit (47 kDa); involved in promoter binding and RNA polymerase II transcription initiation
YPL169C	*MEX67*	0.22	0.22	0.29	**0.24**	Nuclear transport	Poly(A)RNA binding protein involved in nuclear mRNA export; component of the nuclear pore; ortholog of human TAP
YPL210C	*SRP72*	0.19	0.20	0.23	**0.21**	Protein targeting	Core component of the signal recognition particle (SRP); the SRP is a ribonucleoprotein (RNP) complex that functions in targeting nascent secretory proteins to the endoplasmic reticulum (ER) membrane
YPR034W	*ARP7*	0.16	0.35	0.25	**0.25**	Chromatin organization	Component of both the SWI/SNF and RSC chromatin remodeling complexes; actin-related protein involved in transcriptional regulation
YPR082C	*DIB1*	1.00	1.48	0.81	**1.10**	mRNA processing	17-kDa component of the U4/U6aU5 tri-snRNP; plays an essential role in pre-mRNA splicing; human ortholog TXNL4A (the human U5-specific 15-kDa protein) complements yeast dib1 null mutant
