# Supplementary material for: Systematic Identification of Essential Genes Required for Yeast Cell Wall Integrity: Involvement of the RSC Remodelling Complex
Source: J Fungi (Basel). 2022 Jul 8;8(7):718. doi: 10.3390/jof8070718 (PMC9323250; doi:10.3390/jof8070718)
Supplement: Supplementary file 1 [file jof-08-00718-s001.zip › Supplemental Table S5.pdf]

Table S5. Mutant strains that exhibit increased basal Mlp1-GFP fluorescence respect the wild-type levels under non-stress conditions. For each mutant strain, data from three independent experiments (exp) and their corresponding mean value are shown.

Mutated ORF	Mutated gene	High Basal Mlp1-GFP (exp 1)	High Basal Mlp1-GFP (exp 2)	High Basal Mlp1-GFP (exp 3)	Mean	Biological process	Description from the *Saccharomyces Genome Database*
YAL034W-A	*MTW1*	6.6	2.6	4.7	**4.6**	Chromosome segregation	Essential component of the MIND kinetochore complex; joins kinetochore subunits contacting DNA to those contacting microtubules; critical to kinetochore assembly; complex consists of Mtw1p Including Nnf1p-Nsl1p-Dsn1p (MIND)
YBL020W	*RFT1*	6.6	3.1	3.5	**4.4**	Protein glycosylation	Membrane protein required for translocation of Man5GlcNac2-PP-Dol; required for translocation of Man5GlcNac2-PP-Dol from the cytoplasmic side to the lumenal side of the ER membrane but is not the flippase; mutation is suppressed by expression of human p53 protein; essential gene
YBR002C	*RER2*	3.6	3.0	3.2	**3.3**	Lipid metabolism	Forms the dehydrodolichyl diphosphate syntase (DDS) complex with NUS1; major enzyme of polyprenol synthesis in both the endoplasmic reticulum (ER) and in lipid droplets; participates in ER protein sorting; human ortholog DHDDS functionally complements the heat sensitive growth defect of a ts allele, and is associated with retinitis pigmentosa
YBR070C	*ALG14*	3.9	4.7	8.6	**5.7**	Protein glycosylation	Component of UDP-GlcNAc transferase; required for second step of dolichyl-linked oligosaccharide synthesis; anchors catalytic subunit Alg13p to ER membrane; similar to bacterial and human glycosyltransferases; both human homologs ALG13 and ALG14 are required to complement yeast alg14 mutant
YBR196C	*PGI1*	8.7	3.7	3.3	**5.2**	Carbohydrate metabolism	Glycolytic enzyme phosphoglucose isomerase; catalyzes the interconversion of glucose-6-phosphate and fructose-6-phosphate; required for cell cycle progression and completion of the gluconeogenic events of sporulation
YBR254C	*TRS20*	3.2	1.7	4.2	**3.0**	Golgi vesicle transport	Core component of transport protein particle (TRAPP) complexes I-III; TRAPPs are multimeric guanine nucleotide-exchange factors for GTPase Ypt1p, regulating ER-Golgi traffic (TRAPPI), intra-Golgi traffic (TRAPPII), endosome-Golgi traffic (TRAPPII and III) and autophagy (TRAPPIII); mutation leads to defects in endocytic recycling, block in sporulation/meiosis; mutations in human homolog TRAPPC2 cause spondyloepiphyseal dysplasia tarda, TRAPPC2 can complement yeast null mutant
YDL029W	*ARP2*	11.7	4.9	6.3	**7.6**	Cytoskeleton organization	Essential component of the Arp2/3 complex; Arp2/3 is a highly conserved actin nucleation center required for the motility and integrity of actin patches; involved in endocytosis and membrane growth and polarity; required for efficient Golgi-to-ER trafficking in COPI mutants

YDR166C	*SEC5*	4.0	3.7	4.2	**3.9**	Golgi vesicle transport	Essential 107kDa subunit of the exocyst complex; the exocyst mediates polarized targeting and tethering of post-Golgi secretory vesicles to active sites of exocytosis at the plasma membrane prior to SNARE-mediated fusion; involved in assembly of the exocyst complex; required with Sec3p for ER inheritance where it promotes anchoring of the cortical ER at the bud tip
YDR434W	*GPI17*	4.2	4.2	1.9	**3.4**	GPI biosynthesis	Transmembrane protein; subunit of the glycosylphosphatidylinositol transamidase complex that adds GPIs to newly synthesized proteins; human PIG-S homolog
YEL002C	*WBP1*	9.2	5.6	3.1	**5.9**	Protein glycosylation	Beta subunit of the oligosaccharyl transferase glycoprotein complex; required for N-linked glycosylation of proteins in the endoplasmic reticulum; human homolog DDOST can complement yeast growth defect during down-regulation of yeast gene
YER003C	*PMI40*	3.2	3.1	2.9	**3.0**	Protein glycosylation	Mannose-6-phosphate isomerase; catalyzes the interconversion of fructose-6-P and mannose-6-P; required for early steps in protein mannosylation
YER008C	*SEC3*	5.6	4.5	5.0	**5.1**	Golgi vesicle transport	Subunit of the exocyst complex; the exocyst mediates polarized targeting and tethering of post-Golgi secretory vesicles to sites of exocytosis prior to SNARE-mediated fusion; PtdIns[4,5]P2-binding protein that localizes to exocytic sites in a Rho1p-dependent, actin-independent manner, targeting and anchoring the exocyst to the plasma membrane with Exo70p; direct GTP Rho1p effector; required for ER inheritance; relocalizes away from bud neck upon DNA replication stress
YFL005W	*SEC4*	6.5	3.9	4.4	**4.9**	Golgi vesicle transport	Rab family GTPase; essential for vesicle-mediated exocytic secretion and autophagy; associates with the exocyst component Sec15p and may regulate polarized delivery of transport vesicles to the exocyst at the plasma membrane
YFL038C	*YPT1*	4.3	3.1	4.0	**3.8**	Golgi vesicle transport	Rab family GTPase; involved in the ER-to-Golgi step of the secretory pathway; complex formation with the Rab escort protein Mrs6p is required for prenylation of Ypt1p by type II protein geranylgeranyltransferase (Bet2p-Bet4p); binds to unspliced HAC1 mRNA; regulates the unfolded protein response (UPR) by promoting the decay of HAC1 RNA; localizes to the early Golgi, the transitional Golgi and ER membranes, pre-autophagosomal structures, and cytoplasmic vesicles
YFL045C	*SEC53*	5.0	1.3	3.1	**3.1**	Protein glycosylation	Phosphomannomutase; involved in synthesis of GDP-mannose and dolichol-phosphate-mannose; required for folding and glycosylation of secretory proteins in the ER lumen
YGL225W	*VRG4*	3.0	2.6	3.2	**3.0**	Protein glycosylation	Golgi GDP-mannose transporter; regulates Golgi function and glycosylation in Golgi; VRG4 has a paralog, HVG1, that arose from the whole genome duplication

YGL233W	*SEC15*	7.6	6.4	6.8	**6.9**	Golgi vesicle transport	Essential 113 kDa subunit of the exocyst complex; the exocyst mediates polarized targeting and tethering of post-Golgi secretory vesicles to active sites of exocytosis prior to SNARE-mediated fusion; interacts with and functions as a downstream effector of active, GTP-bound Sec4p, a Rab family GTPase
YHR107C	*CDC12*	9.5	4.4	2.0	**5.3**	Cell cycle	Component of the septin ring that is required for cytokinesis; septins are GTP-binding proteins that assemble into rod-like hetero-oligomers that can associate with other rods to form filaments; septin rings at the mother-bud neck act as scaffolds for recruiting cell division factors and as barriers to prevent diffusion of specific proteins between mother and daughter cells
YHR188C	*GPI16*	5.2	8.0	5.6	**6.3**	GPI biosynthesis	Subunit of the glycosylphosphatidylinositol transamidase complex; transmembrane protein; adds GPIs to newly synthesized proteins; human PIG-Tp homolog
YIL046W	*MET30*	4.3	5.3	4.9	**4.8**	Cell cycle	F-box protein containing five copies of the WD40 motif; controls cell cycle function, sulfur metabolism, and methionine biosynthesis as part of the ubiquitin ligase complex; interacts with and regulates Met4p, localizes within the nucleus; dissociation of Met30p from SCF complex in response to cadmium stress is regulated by Cdc48p
YIL109C	*SEC24*	3.6	3.5	3.9	**3.6**	Golgi vesicle transport	Component of the Sec23p-Sec24p heterodimer of the COPII vesicle coat; required for cargo selection during vesicle formation in ER to Golgi transport; homologous to Sfb3p; SEC24 has a paralog, SFB2, that arose from the whole genome duplication
YKR037C	*SPC34*	5.0	8.2	8.1	**7.1**	Chromosome segregation	Essential subunit of the Dam1 complex (aka DASH complex); complex couples kinetochores to the force produced by MT depolymerization thereby aiding in chromosome segregation; also localized to nuclear side of spindle pole body
YLL050C	*COF1*	5.8	7.7	8.0	**7.2**	Cytoskeleton organization	Cofilin, involved in pH-dependent actin filament depolarization; binds both actin monomers and filaments and severs filaments; involved in the selective sorting, export of the secretory cargo from the late golgi; genetically interacts with pmr1; thought to be regulated by phosphorylation at SER4; ubiquitous and essential in eukaryotes
YLR166C	*SEC10*	10.3	8.4	9.7	**9.5**	Golgi vesicle transport	Essential 100kDa subunit of the exocyst complex; the exocyst mediates polarized targeting and tethering of post-Golgi secretory vesicles to active sites of exocytosis at the plasma membrane prior to SNARE-mediated fusion
YLR229C	*CDC42*	4.0	4.7	18.4	**9.0**	Signaling	Small rho-like GTPase; essential for establishment and maintenance of cell polarity; plays a role late in cell fusion via activation of key cell fusion regulator Fus2p; mutants have defects in the organization of actin and septins; human homolog CDC42 can complement yeast cdc42 mutant

YLR459W	*GAB1*	7.4	5.2	6.8	**6.5**	GPI biosynthesis	GPI transamidase subunit; involved in attachment of glycosylphosphatidylinositol (GPI) anchors to proteins; may have a role in recognition of the attachment signal or of the lipid portion of GPI
YML130C	*ERO1*	6.2	3.9	3.7	**4.6**	Protein folding	Thiol oxidase required for oxidative protein folding in the ER; essential for maintaining ER redox balance; feedback regulated via reduction and oxidation of regulatory bonds; reduced Pdi1p activates Ero1p by direct reduction of Ero1p regulatory bonds; depletion of thiol substrates and accumulation of oxidized Pdi1p results in inactivation of Ero1p by both Pdi1p-mediated oxidation and autonomous oxidation of Ero1p regulatory bonds; ero1-1 mutation complemented by human ERO1L
YMR149W	*SWP1*	4.6	2.9	2.6	**3.4**	Protein glycosylation	Delta subunit of the oligosaccharyl transferase glycoprotein complex; complex is required for N-linked glycosylation of proteins in the endoplasmic reticulum
YMR200W	*ROT1*	8.3	6.8	8.8	**8.0**	Protein folding	Molecular chaperone involved in protein folding in ER; mutation causes defects in cell wall synthesis and lysis of autophagic bodies, suppresses tor2 mutations, and is synthetically lethal with kar2-1 and with rot2 mutations; involved in N-linked glycosylation and O-mannosylation; transmembrane helix Ser250 is essential for Rot1p to interact with other membrane components and exert its functional role, avoiding exposure of Ser H-bonding group at lipid-exposed surface
YMR281W	*GPI12*	4.8	2.1	2.2	**3.0**	GPI biosynthesis	ER membrane protein involved in the second step of GPI anchor assembly; the second step is the de-N-acetylation of the N-acetylglucosaminylphosphatidylinositol intermediate; functional homolog of human PIG-Lp; GPI stands for glycosylphosphatidylinositol
YNL048W	*ALG11*	3.1	3.2	2.8	**3.1**	Protein glycosylation	Alpha-1,2-mannosyltransferase; catalyzes sequential addition of the two terminal alpha 1,2-mannose residues to the Man5GlcNAc2-PP-dolichol intermediate during asparagine-linked glycosylation in the ER
YNL158W	*PGA1*	6.2	10.8	22.0	**13.0**	GPI biosynthesis	Essential component of GPI-mannosyltransferase II; complex is responsible for second mannose addition to GPI precursors as a partner of Gpi18p; required for maturation of Gas1p and Pho8p; has synthetic genetic interactions with secretory pathway genes
YNL171C	*YNL171C*	5.0	6.0	5.4	**5.4**	Unknown	Dubious open reading frame; unlikely to encode a functional protein, based on available experimental and comparative sequence data
YNR035C	*ARC35*	11.1	4.3	3.4	**6.3**	Cytoskeleton organization	Subunit of the ARP2/3 complex; ARP2/3 is required for the motility and integrity of cortical actin patches; required for cortical localization of calmodulin
YOR103C	*OST2*	5.8	6.5	4.9	**5.7**	Protein glycosylation	Epsilon subunit of the oligosaccharyltransferase complex; located in the ER lumen; catalyzes asparagine-linked glycosylation of newly synthesized proteins

YOR181W	*LAS17*	3.3	3.1	4.3	**3.6**	Cytoskeleton organization	Actin assembly factor; C-terminal WCA domain activates Arp2/3 complex-mediated nucleation of branched actin filaments, polyproline domain nucleates actin filaments independent of Arp2/3; mutants are defective in endocytosis, bud site selection, cytokinesis; human homolog WAS (Wiskott-Aldrich Syndrome) implicated in severe immunodeficiency; human WAS complements yeast null mutant, but only in presence of WIPF1, which mediates localization of WAS to cortical patches
YOR254C	*SEC63*	4.7	3.6	4.3	**4.2**	Protein targeting	Essential subunit of Sec63 complex; with Sec61 complex, Kar2p/BiP and Lhs1p forms a channel competent for SRP-dependent and post-translational SRP-independent protein targeting and import into the ER; other members are Sec62p, Sec66p, and Sec72p
YOR341W	*RPA190*	4.8	3.0	3.1	**3.6**	Transcription from RNA pol I	RNA polymerase I largest subunit A190
YPL082C	*MOT1*	4.4	3.3	3.7	**3.8**	Transcription from RNA pol II	Essential protein involved in regulation of transcription; removes Spt15p (TBP) from DNA via its C-terminal ATPase activity; may have a role in ensuring that soluble TBP is available to bind TATA-less promoters; forms a complex with TBP that binds TATA DNA with high affinity but with altered specificity; the Mot1p-Spt15p-DNA ternary complex contains unbent DNA; coregulates transcription with Spt16p through assembly of preinitiation complex and organization of nucleosomes
YPL218W	*SAR1*	3.4	3.2	3.2	**3.3**	Golgi vesicle transport	ARF family GTPase; component of the COPII vesicle coat; required for transport vesicle formation during ER to Golgi protein transport; lowers membrane rigidity aiding vesicle formation; localizes to ER-mitochondrial contact sites where it enhances membrane curvature, thereby reducing contact size via its N-terminal amphipathic helix; regulates mitochondrial fission and fusion dynamics
YPR183W	*DPM1*	3.0	3.0	4.0	**3.3**	Protein glycosylation	Dolichol phosphate mannose (Dol-P-Man) synthase of ER membrane; catalyzes formation of Dol-P-Man from Dol-P and GDP-Man; required for biosynthesis of glycosyl phosphatidylinositol (GPI) membrane anchor, as well as O-mannosylation and protein N- and O-linked glycosylation; human homolog DPM1 can complement yeast mutant strains
